# Human Health Effects of Oral Exposure to Chromium: A Systematic Review of the Epidemiological Evidence

**DOI:** 10.3390/ijerph21040406

**Published:** 2024-03-27

**Authors:** Eleni Sazakli

**Affiliations:** Lab of Public Health, Medical School, University of Patras, GR 26504 Patras, Greece; elsazak@upatras.gr

**Keywords:** chromium, hexavalent chromium, oral exposure, epidemiological studies, biomonitoring studies, health effects, prenatal exposure

## Abstract

The toxicity and carcinogenicity of hexavalent chromium via the inhalation route is well established. However, a scientific debate has arisen about the potential effects of oral exposure to chromium on human health. Epidemiological studies evaluating the connection between ingested chromium and adverse health effects on the general population are limited. In recent years, a wealth of biomonitoring studies has emerged evaluating the associations between chromium levels in body fluids and tissues and health outcomes. This systematic review brings together epidemiological and biomonitoring evidence published over the past decade on the health effects of the general population related to oral exposure to chromium. In total, 65 studies were reviewed. There appears to be an inverse association between prenatal chromium exposure and normal fetal development. In adults, parameters of oxidative stress and biochemical alterations increase in response to chromium exposure, while effects on normal renal function are conflicting. Risks of urothelial carcinomas cannot be overlooked. However, findings regarding internal chromium concentrations and abnormalities in various tissues and systems are, in most cases, controversial. Environmental monitoring together with large cohort studies and biomonitoring with multiple biomarkers could fill the scientific gap.

## 1. Introduction

Chromium [Cr] is a transition metal that constitutes the 21st most abundant element in the Earth’s crust, with an overall concentration of 125 mg/kg [1], and occurs in nature mainly in two oxidation states, +3 and +6. The primary species of Cr found in the environment, trivalent chromium [Cr(III)], is largely of geological origin and is less soluble and mobile than the second most stable oxidation state, hexavalent Cr, Cr(VI). Cr(VI) rarely occurs naturally and is mainly emitted into the environment as a result of anthropogenic activities [2]. The leading industrial application of Cr(VI) is the manufacturing of stainless steel, which comprises approximately 90% of all chromite ore use. In addition, Cr(VI) compounds are widely used as corrosion inhibitors, in the manufacturing of pigments, for metal finishing and chrome plating, in leather tanning, and as wood preservatives [3]. In environmental compartments, the speciation of chromium is governed by the prevailing physicochemical factors (pH, redox potential, presence and levels of chromium species and reducing or oxidizing agents, etc.), and inter-conversion of the two species can occur [4]. Notably, naturally derived Cr(VI) may occur within ophiolite-related aquifers as the result of oxidation of Cr(III) to Cr(VI) by electron acceptors such as Mn-oxides [5]. All in all, Cr(VI) compounds may be present in the aquatic environment due to direct discharge from industrial operations, wet and dry deposition, leaching from soils, and weathering of Cr-containing rocks [4]. 

Not all Cr(VI) compounds are soluble in water. Water-insoluble chromate pigments like BaCrO_4_ or PbCrO_4_ or sparingly soluble pigments like ZnCrO_4_ are not important contributors in the aquatic chromium cycle; however, they have been implicated in the induction of lung cancers, as it has been reported that inhalation of water-insoluble or sparingly soluble industrial chromium respirable particles, such as those released in the air directly from industrial processes or fugitive emissions from improperly disposed chromium materials, are the ones that are carcinogenic [4,6,7]. In contrast, highly water-soluble compounds like Na_2_CrO_4_ and K_2_CrO_4_ (solubility ≥ 500 g/L) are the compounds that predominate in the aquatic environment. The ionic species of Cr(VI) in basic and neutral conditions is chromate (CrO_4_^−2^), whereas as the pH decreases, the concentration of hydrochromate increases (HCrO_4_^−^). At very low pH and high concentrations of CrO_4_^−2^, chromates are dimerized to dichromate species (Cr_2_O_7_^−2^), which is a strong oxidizing agent, in contrast to chromates, which are poor oxidizing agents [4].

IARC has classified Cr(VI) compounds as carcinogenic to humans (Group 1), as more than 50 epidemiological studies have provided information on cancer risks from Cr(VI) [2]. Most epidemiological studies concern exposure in occupational settings, where, in theory, concentrations are high and exposure occurs repeatedly. Many agencies have set limits on Cr(VI) levels in order to protect human health. Cr(VI) has recently been added to the EU Carcinogens and Mutagens Directive (CMD), with a binding occupational limit value of 0.005 mg Cr(VI)/m^3^ (5 μg/m^3^) to enter into force starting in 2025. In the US, ACGIH proposed in 2018 a threshold limit value (TLV) of 0.2 μg/m^3^, which is based on non-cancer (lung) effects. The general population residing in the vicinity of anthropogenic sources of Cr(VI) can be exposed through inhalation of ambient air or by ingestion of contaminated drinking water. Most of the chromium ingested with food is Cr(III), which is readily taken up by plants and enters the food chain [2,8].

Nevertheless, the potential health effects due to oral consumption of Cr(VI) via drinking water is an issue that has yet to be clarified. After oral administration, Cr(VI) encounters saliva and acidic gastric fluid, where rapid and efficient reduction of Cr(VI) to Cr(III) takes place by extracellular reductants such as ascorbate, glutathione, or cysteine [9]. The octahedral structure of Cr(III) does not allow for entry to cells. Conversely, Cr(VI) can enter cells via the anionic transport system due to its structural similarity as an oxyanion to sulfates and phosphates [10]. Intracellularly, ROS scavengers such as glutathione and ascorbic acid will bind Cr(VI) and reduce it to Cr(III). Intracellular reduction processes generate free radicals, and due to scavenger imbalance, ROS can accumulate and cause damage. Furthermore, although Cr(VI) cannot bind to DNA or other macromolecules in cells, its metabolic intermediates (Cr(V), Cr(IV), and the final product Cr(III)) are highly reactive and readily form Cr-DNA adducts [11]. There is a longstanding scientific debate as to whether the extracellular reduction of Cr(VI) to Cr(III), which is a detoxification process, has the capacity and time to reduce ingested Cr(VI) completely [9,12], or instead, even a small amount of Cr(VI) escapes extracellular reduction as a result of the pseudo first-order kinetics of the reduction by gastric juice under fasting conditions and Cr(VI) enters cells [10,13] In that case, it will be reduced intracellularly, promoting the chain reactions that form reactive oxygen species and Cr-macromolecules adducts, which is an activation process. 

Another topic that has concerned the scientific community is the beneficial action of Cr(III). For a long time, Cr(III) has been popularized as essential to the efficacy of insulin in regulating carbohydrate, lipid, and protein metabolism [14]. A proposed mechanism is that Cr(III) is transported by an oligo-peptide, chromodulin, in response to an insulin-mediated chromic ion influx, and in turn, the metal-transporting oligopeptide binds to the insulin-stimulated receptor by activating the receptor’s tyrosine kinase activity [15]. Cr(III) has also been reported to improve insulin sensitivity by reducing hepatic and muscle intracellular lipid accumulation and/or activation of glucose transporter 4 trafficking [16]. Nevertheless, any consistent dose–response relationship between Cr(III) and beneficial health outcomes in humans has yet to be established, and the European Food Safety Authority’s expert scientific panel currently does not support continued essential classification [14].

Occupational exposure to Cr(VI) through biomonitoring studies has recently been reviewed [17]. Possible health effects on the general population that may be induced by ingestion of hexavalent chromium remain unknown, even though a lot of research has been conducted. The aim of this systematic review is to compile the scientific data to date and elucidate the main findings concerning health outcomes on the general population due to oral exposure to Cr(VI). 

## 2. Methodology

### 2.1. Study Eligibility

The PEO question framework was used to identify the three concepts: population, exposure, and outcome. Specified criteria were defined for studies to be eligible for inclusion in the review. Studies should have been published within the past decade (2013–2023). The eligible population included both children and adults who should not have been occupationally exposed to chromium. Exposure to Cr(VI) should have been via the oral route; therefore, studies on inhalation exposure to chromium were excluded. No exclusion criteria were established for adverse health effects. Therefore, health outcomes included mortality; adverse birth and neonatal effects; altered hematological and biochemical parameters; mental health conditions; cardiovascular, renal, and liver function; diabetes; obesity; and cancer. Because only two studies concerning environmental oral exposure to Cr(VI) and health effects on the general population were published in the last decade, it was decided to extend the time period beyond the last decade and also include relevant epidemiological studies regardless of when they were conducted as long as they involved general populations that were documented to have been exposed to Cr(VI) via the oral route. In addition, biomonitoring studies that investigated health outcomes in relation to Cr concentrations in body fluids (blood, urine) and tissues (hair, toenails) were included, as they provided information on associations between internal Cr levels and effects on health. The design of the studies could be cross-sectional, cohort, birth cohort, ecological, case control, or nested case control within a cohort. Finally, to be eligible for inclusion, studies had to be peer reviewed and published in English. Animal, in vitro, and mechanistic studies were excluded. 

### 2.2. Literature Search

Scientific databases were searched according to Preferred Reporting Items for Systematic Reviews and Meta-Analyses (PRISMA) [18]. The following search terms were used in the PubMed, Scopus, and Web of Science databases: “chromium (chromium* or Cr)” AND “exposure” AND “effect” AND “epidemiological”. Initially, only studies published in the last decade were extracted. In this phase, all biomonitoring studies that were reviewed within were extracted. In a second stage, a new search was performed using the following search terms: “chromium (chromium* or Cr)” AND “oral exposure” AND “effect “AND “epidemiological”, without a publication time restriction, in order to find epidemiological studies that focused on oral exposure of the general population. Inclusion criteria were (a) human studies that examined exposure to Cr(VI) via the oral route in relation to at least one health effect, without a publication time restriction, and (b) biomonitoring studies that examined Cr concentration in body fluids (blood, urine) or tissues (hair, nails) and reported at least one health outcome, published within the last decade (2013–2023). 

**Figure 1:**
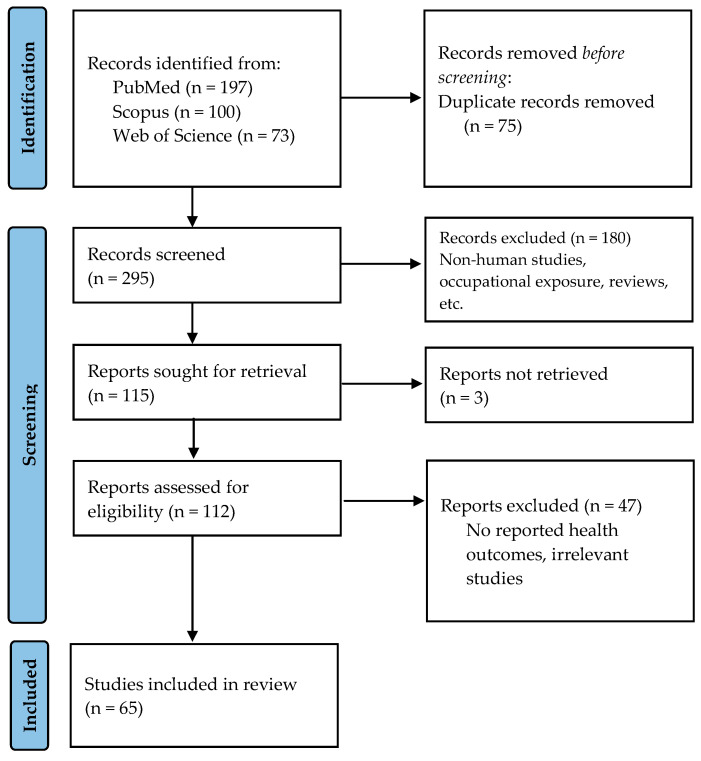
PRISMA flowchart of the study inclusion process.

Titles and abstracts were initially and independently screened by two reviewers according to the eligibility criteria. Selected abstracts, after exclusion of inappropriate studies (non-human studies, occupational exposure, publications in non-English language, reviews) were searched for full-text retrieval. Full manuscripts were subjected to a thorough review and studies without reported health effects and mechanistic or in vitro studies were excluded. Thereafter, data were extracted from the 65 eligible studies (Figure 1). 

Quality of the extracted studies was assessed through the Newcastle–Ottawa scale [19], with scoring on three categories: selection of the study participants, comparability of the groups, and ascertainment of either the exposure or outcome of interest for case control or cohort studies, respectively. For cross-sectional studies, the modified Newcastle–Ottawa scale was used, as mentioned in [20]. Stars were awarded so that the highest-quality studies were scored with nine stars (for cohort and case control studies) and ten stars for cross-sectional studies.

## 3. Results

The initial concept of this review was to identify epidemiological studies on health effects in the general population related to oral exposure to Cr(VI) over the past decade (2013–2023). Since only two studies were extracted, the time span of the literature search was extended beyond any time limit (Section 3.1 and Table 1).

### 3.1. Oral Exposure to Chromium and Health Effects on the General Population

In this review, epidemiological studies that evaluated the oral exposure of the general population to Cr(VI) and its associations with adverse health effects were included. Worth mentioning is that the concentration of Cr in uncontaminated water is very low, about 1–10 μg/L in surface and shallow groundwaters [11,21]. Areas with high concentrations of Cr(VI) in drinking water are fortunately relatively rare, and thus four areas were identified (Table 1). The most studied area concerns the suburbs of Jinzhou in Liaoning province in China, where a set of ecological mortality studies [22,23,24,25] investigated the association of cancer mortality (lung, stomach, etc.) with prolonged oral consumption of water contaminated with Cr(VI). Extreme pollution of the area was caused by a ferrochromium plant in the early 1960s. Cr(VI) concentrations in groundwater and wells ranged from 0.002 up to 20.0 mg/L in 1966, and after the adoption of control measures beginning in 1967, the concentration of Cr(VI) in groundwater fell to the range of 0.01–0.05 mg/L by 1974 [22]. The first study [22] reported increased tumor mortality rates for the exposed population; however, it provided sparse information on the magnitude and duration of Cr exposure, as well as on unexposed populations. The following research [23], which withdrew the original association found in [22], was eventually retracted by the journal editor due to “financial and intellectual input to the paper by outside parties that had not been disclosed”. A later re-analysis [24] confirmed increased stomach cancer mortality for the areas with polluted water (rate ratio, RR = 1.82, 95% CI: 1.11–2.91)—a finding, however, that was not verified in a subsequent re-examination of the same data, which used a smaller number of controls from nearby areas without Cr(VI) in groundwater [25]. A detailed chronology of the key events regarding cancer mortality risks in the population orally exposed to Cr(VI) in Liaoning province in China can be found in [26]. A more recent cross-sectional study [27] conducted in this study area found increased levels of oxidative stress parameters (malondialdehyde, glutathione peroxidase, catalase) and of a DNA damage biomarker (8-hydroxy-2 deoxyguanosine, 8-OHdG) and lower activity of serum SOD (superoxide dismutase) in the exposed group compared to the unexposed group (*p* < 0.0001). All models were adjusted for gender, age, smoking status, alcohol consumption, personal income, and educational level.

The second area, with water Cr(VI) concentrations up to 400 times higher than the guideline value of 50 μg/L, was Kanpur, one of the most industrialized cities in India, with many tanneries and Cr-processing industries. A cross-sectional retrospective study assessed self-reported health problems in the exposed population and documented higher odds of gastrointestinal problems and skin abnormalities (Table 1). Residents from affected communities had significantly higher red blood cell counts (RBCs), lower mean corpuscular volume (MCV), and fewer platelets than matched controls [28].

The third area was in Greece, in Voiotia prefecture, where Cr(VI) pollution of groundwater, which was the source of drinking water at the time, was attributed mainly to geological origin, and to some extent to industrial pollution. The issue came to light in around 2009 and immediate actions were taken; thus, in most cases the water supply was diverted to receive surface, Cr-free water [29]. This population was exposed to relatively high levels, ranging from non-detectable to up to 196.0 μg/L, in the water supply for many years and served as the study population in three studies [29,30,31]. In the cross-sectional retrospective study [29], a personal lifetime dose of Cr exposure was calculated for each participant and associated with blood and hair Cr concentrations (Table 1). Hematologic and biochemical alterations were associated with either Cr exposure dose, blood Cr, or hair Cr (Table 1 and Table 2). A morbidity cohort study [30] in the same area assessed exposure using years of residence. In men, higher odds of lower urinary tract symptoms were observed as length of residence increased, whereas odds of genitourinary infections were statistically significantly higher for those having lived for more than 15 years in the area [30]. Finally, an ecological mortality study [31] found extremely high standardized mortality ratios (SMRs) for primary liver cancer and lung cancer in both sexes and for kidney and other genitourinary cancers only in women. This study [31] was questioned about exposure assessment (whether it was a worst-case scenario) and the lack of significant association between stomach cancer and Cr exposure [32]. 

The final study area was in California, USA, and involved residential exposure to Cr(VI) from the Hinkley plant [33]. An ecological mortality study did not find significantly higher mortality rates in exposed areas compared to unexposed areas; however, mortality rates were only age-adjusted, and analyses of geographic areas were based on postal zip codes [33].

The above epidemiological studies attempted to reveal the possible adverse health effects in populations exposed to various levels of Cr(VI) via the oral route. They followed different study designs and investigated different health outcome measures. Therefore, a direct comparison of their results is not possible. However, oral exposure to Cr(VI) seems to be associated with alterations in some hematological and biochemical parameters [28,29]. Exposed people also appear to have higher odds of developing gastrointestinal, skin, and urinary symptoms diagnosed or evaluated by medical staff, albeit self-reported [28,30]. Finally, as shown by the ecological studies conducted in China [22,23,24,25] and in Greece [31], there are indications of increased mortality rates from stomach and urinary system cancers. Even though Cr(VI) concentrations in groundwater have been reported at truly high levels in China, exposure assessment was not carried out in a systematic and representative manner, nor were potential confounders considered, increasing the uncertainty of the calculated mortality rates and ratios. In contrast, exposure assessment in the Greek population was based on longitudinal systematic monitoring of surface water and groundwater carried out by various agencies. However, the large difference in exposure levels between China and Greece is not reflected in the estimated mortality rates, raising doubts about the consistency and repeatability of the findings. It is certain that no safe conclusion can be drawn regarding oral Cr(VI) exposure and cancer mortality, as the findings are controversial. Most of these studies have an ecological design and inherently suffer from information loss. Associations and risks were estimated at the community level and individual exposure data are lacking. Therefore, an exposure–response relationship is not evident and exposure misclassification cannot be ruled out. In only one study [29], oral exposure to Cr(VI) was correlated with internal Cr levels and changes in hematological/biochemical parameters, while in the other cross-sectional study [28], exposure was assessed at the community level, allowing for probable differential misclassification bias. The challenge is big, and the knowledge gaps require large retrospective cohort studies that can be conducted on these chronically exposed populations. Considerable effort is needed to quantify adverse effects, which may range from subtle physiological and biochemical changes to symptoms of illness, clinically diagnosed disease, or, finally, death. Only with a well-designed and controlled exposure assessment strategy, followed by a structured methodology and evaluation of health outcomes and appropriate adjustment for potential confounding factors and effect modifiers, can the relationship of oral exposure to Cr(VI) to health damage be discovered. 

### 3.2. Associations of Cr Biomarkers and Adverse Health Effects during Lifetime

#### 3.2.1. Prenatal Cr Exposure and Fetal Development 

Exposure to toxic substances causes adverse health effects in all life stages; however, when exposure occurs during critical developmental windows, it may become detrimental to the future of the person. The field of epigenetics, i.e., the interaction of genes with their environment during development, gives insight into how chemical modifications of the genome directly influence the production of proteins that may alter the phenotype of an organism [34]. According to the developmental origin of health and disease (DOHaD) hypothesis, harmful exposures that occur during the pre- and perinatal period, while tissues and organs are developing, may influence development and increase the risk of disease later in life through multiple mechanisms [35]. Chromium has been reported to cross the placental barrier, and consequently, it may pose a significant hazard to a fetus during development.

**Table 1:** Summary of the studies on environmental Cr(VI) oral exposure of the general population.

Study Characteristics (Area, Time, Population)	Study Design	Exposure Levels	Key Findings of Health Outcomes	Ref.
China, Jinzhou area, 1970–1978	Ecological mortality	Drinking water Cr(VI): up to 20,000 μg/L	Malignant tumor mortality rates: 71.89–92.66/100,000 vs. 65.40/100,000 (control)	[22]
China, Jinzhou area, 1965–1978	Ecological mortality	Average Cr(VI) well-water concentration <0.001–20.0 mg/L	All cancer deaths: RR = 1.13, (95% CI: 0.86, 1.46) **Stomach cancer deaths: RR = 1.82, (95% CI: 1.11, 2.91)** Lung cancer deaths: RR = 1.15, (95% CI: 0.62, 2.07)	[24]
China, Jinzhou area, 1965–1978	Ecological mortality	Average Cr(VI) well-water concentration 0.004–10.5 mg/L	All cancer deaths: RR = 1.10, (95% CI: 0.80, 1.51) Stomach cancer deaths: RR = 1.22, (95% CI: 0.74, 2.01) Lung cancer deaths: RR = 1.76, (95% CI: 0.78, 3.98)	[25]
China, Liaoning province, villages of Jinzhou city 2016 626 adults: 319 exposed 307 non-exposed	Cross-sectional	Cr in groundwater: 0.002–2.5 mg/L Cr in soil: 20.1–417.1 mg/kg Cr in air: 5.0–82.9 ng/m^3^ Exposure surrogate: duration of residence	**MDA: β = 0.32**, *p* = 0.0001 **CAT: β = 1.60**, *p* < 0.0001/increase with length of residence for age <18 at first exposure **SOD: β = −14.73**, *p* < 0.0001 **GSH-Px: β = 45.66**, *p* < 0.0001/increase with length of residence for age >18 at first exposure **8-OHdG (log): β = 0.09**, *p* = 0.0075/increase with length of residence for age <18 at first exposure	[27]
India, Pradesh Uttar, Kanpur 186 exposed 230 non-exposed	Cross-sectional retrospective	Drinking water Cr(VI): 20,000 μg/L	**Gastrointestinal symptoms: OR = 3.1, (95% CI: 1.50, 6.39)** (men), **OR = 2.44, (95% CI: 1.32, 4.52)** (women) **Skin symptoms: OR= 3.5 (95% CI 1.41, 8.58)** (men), **OR = 6.57, (95% CI 2.64, 16.32)** (women) **Ocular complaints: OR= 3.5 (95% CI 1.22, 9.79)** (men) **Urinary complaints: OR= 3.1 (95% CI 1.08, 8.87)** (women)	[28]
India, Pradesh Uttar, Kanpur 143 exposed 70 non-exposed	Cross-sectional retrospective	Drinking water Cr(VI): 20,000 μg/L	**RBC count: 5.55 ± 1.39 (exposed men) vs. 4.28 ± 0.69 (control men), *p* < 0.001** **RBC count: 5.67 ± 1.26 (exposed women) vs. 3.89 ± 0.71 (control women), *p* < 0.001** **MCV: 78.56 ± 9.18 (exposed men) vs. 85.38 ± 7.89 (control men), *p* < 0.001** **PLT: 116.2 ± 42.9 (exposed men) vs. 190.3 ± 59.3 (control men), *p* < 0.001** **PLT: 120.2 ± 56.5 (exposed women) vs. 228.4 ± 76.9 (control women), *p* < 0.001** No association with total leucocyte count	[28]
Greece, Voiotia prefecture 2012–2014 122 currently exposed 115 exposed in past 67 non-exposed	Cross-sectional retrospective	Lifetime Cr exposure dose: 3738.0 μg/kg BW (range: 26.1–21,574.7) (currently exposed) Lifetime Cr exposure dose: 1654.6 μg/kg BW (range: 8.6–29,281.1) (exposed in the past) Lifetime Cr exposure dose: 307.1 μg/kg BW (range: 54.0–3736.7) (non-exposed)	**Significant associations with Cr exposure dose (ln)** **Cr-B: β = 0.134, *p* = 0.023/Cr-H(ln): β = 0.226, *p* < 0.001** **SBP: β = 0.142, *p* = 0.010/DBP: β = 0.116, *p* = 0.042** **Hb: β = −0.093, *p* = 0.041/hematocrit: β = −0.094, *p* = 0.048** **TG(ln): β = 0.144, *p* = 0.009/HDL: β = −0.113, *p* = 0.034** **Sodium: β = −0.145, *p* = 0.011/Calcium: β = 0.117, *p* = 0.044** **Alkaline phosphatase: β = 0.120, *p* = 0.035** **Amylase: β = 0.159, *p* = 0.005** **Albumin: β = 0.213, *p* < 0.001/TP: β = 0.144, *p* = 0.012** **IL-12 (ln): β = 0.308, *p* = 0.012**	[29]
Greece, Voiotia, Oinofyta 2010–2011 1181 exposed (1/3 of the total population)	Morbidity Cohort	Exposure surrogate: duration of residence Mean: 17.6 years of residence	**Lower urinary tract symptoms: OR = 1.11, *p* = 0.050 in men** **Urogenital infections: OR = 1.91, *p* = 0.049 for >15 years residence**	[30]
Greece, Voiotia, Oinofyta 1999–2009 5842 exposed	Ecological mortality	Drinking water Cr(VI): range 8–156 μg/L (N = 106)	**Primary liver cancer: SMR= 11.04, (95% CI: 4.05, 24.03)** **Kidney and genitourinary organ cancers: SMR= 3.68, (95% CI: 1.19, 8.58) in women** **Lung cancer: SMR= 1.45, (95% CI 1.01, 2.03)**	[31]
USA, California, Kettleman, Hinkley, Topock 1989–1998 No data on persons at risk	Ecological mortality	No data about Cr(VI) concentrations or duration of exposure	Lung cancer deaths: RR = 1.03, (95% CI: 0.90, 1.17) All cancer deaths: RR = 0.93, (95% CI: 0.87, 1.00) All deaths: RR = 0.98, (95% CI: 0.95, 1.02)	[33]

BW: body weight, CAT: catalase, CI: confidence interval, Cr-B: Cr in blood, Cr-H: Cr in hair, DBP: diastolic blood pressure, GSH-Px: glutathione peroxidase, Hb: hemoglobin, HDL: high-density lipoprotein, IL-12: interleukin-12, MCV: mean corpuscular volume, MDA: malondialdehyde, OR: odds ratio, PLT: platelet count, RBC: red blood cell, RR: rate ratio, SBP: systolic blood pressure, SMR: standardized mortality rate, SOD: superoxide dismutase, TG: total triglycerides, 8-OHdG: 8-hydroxy-2′-deoxyguanosine. Significant associations are indicated in bold.

Fifteen biomonitoring studies evaluated the associations of metal concentrations in mothers with adverse birth outcomes in fetuses and neonates (Table 2). Among the most studied outcomes is low birth weight (LBW) [36,37,38,39,40], defined as weight at birth <2500 g, which has been associated with neonatal mortality. Other fetal growth parameters that were examined for their association with maternal urinary, placental, and umbilical cord concentrations of chromium are estimated fetal weight (EFW) [41,42], birth length [38], abdominal circumference (AC) [41], ponderal index [41], gestational age (preterm birth) [40,43,44], and premature rupture of membranes (PROM) spontaneously ahead of the onset of labor [45]. 

Negative associations of Cr concentrations in maternal urine [36,38,41,42] and placenta [40] with estimated fetal weight (EFW) or weight at birth were found in one case control [36], three birth cohorts [40,41,42], and one cross-sectional study [38]. These studies examined 6134 mother–infant pairs in total and all were of good quality regarding the selection of the population, the comparability of the groups, and the assessment of outcome according to the Newcastle–Ottawa Quality Assessment Scale (8–9 stars). Average birth weight ranged from 3278.7 ± 441.4 g [38] to 3357.84 ± 445.01 g [42]. The magnitude of associations found in these studies is presented in Table 2. Indicatively, in the case control study, the risk of low birth weight (LBW) was more than twice as high in Chinese mothers in the highest tertile of urinary Cr levels (≥6.78 μg/g creatinine) compared to those in the lowest tertile (<3.03 μg/g creatinine). Female infants seemed to be more vulnerable than boys in the case control study [36], whereas negative associations found in [41] were more pronounced in male fetuses. In contrast, two other studies, one birth cohort [37] and one cross-sectional [39], which enrolled 1205 pairs of mothers–infants, did not find any association between Cr levels and the birth weight of the infant. An apparent difference in these studies is that Cr concentrations were determined in blood or serum samples collected from the umbilical cord and not in urine samples of the mothers or the placenta. Moreover, the cross-sectional study was conducted in a truly rural area (Canary Islands, Spain) with a very low level of industrialization [39], a fact represented in the determined concentrations of Cr. In all the above studies, final models were controlled for the most significant covariates, including gestational age and sex of the infant (if no sex-stratified analysis was conducted), as well as parity, maternal age, occupation, education, household income, lifestyle of mothers during pregnancy (tobacco and alcohol use), pre-pregnancy body mass index, pregnancy complications (hypertensive disorders in pregnancy and gestational diabetes mellitus), and any apparent congenital malformations.

Birth length [38,40], abdominal circumference (AC) [41], and ponderal index [41] were all negatively associated with maternal urinary [38,41] or placenta Cr levels [40]. 

Higher risk of preterm delivery (<37 weeks of pregnancy) or PROM and a negative association of gestational age with maternal Cr levels were found in two large birth cohorts [44,45], with a total of 12,698 mother–infant pairs. No association was observed in a Chinese nested case control study [43], while a slight increase in gestational age (0.56 weeks) was reported in the INMA birth cohort [40], in which preterm birth was observed in eight newborns (2.4%).

Other health outcomes that were examined and did not reveal any association with maternal Cr levels were neural tube defects (NTDs) [46], congenital heart defects (CHDs) [47], and craniosynostosis (CS) [48]. 

Two studies [49,50] investigated fetal exposure to Cr, among other metals, in relation to health effects in early childhood. Maternal urinary Cr concentration in the third trimester of gestation was associated with a higher risk of allergic rhinitis in childhood at age 4 [49]. In addition, Cr showed a positive association with wheezing and eczema when all the other determined metals were fixed at their medians by Bayesian kernel machine regression (BKMR) analysis. Emotional development, specifically child positive affectivity, was negatively affected by higher Cr burden in maternal hair [50]. The study was conducted in a war zone during the 2014 Gaza war. Surprisingly, maternal post-traumatic stress (PTSD) did not modify the outcome [50]. 

#### 3.2.2. Cr Exposure during Pregnancy and Health Outcomes in the Mother

Pregnancy is a period of rapid physiological changes, accompanied by increased expression of oxidative stress markers and deficiency of antioxidants, which may enhance toxic insults from metals [51,52]. Three studies [51,53,54] examined the associations of Cr body burden with health outcomes in pregnant women during gestation (Table 2). No significant association between Cr levels in maternal plasma during pregnancy and the risk of gestational diabetes mellitus (GDM) was found in a nested case control study [53]. Preeclampsia risk was significantly higher in pregnant women with detectable amounts of Cr (>0.4 μg/L) in urine (HR = 3.48, 95% CI: 1.02–11.8); albeit Cr was detected only in 15.4% of the study population [54]. In another study, Cr was found to be a major contributor (14.7%) to the overall positive association of joint exposure to six metals with maternal anxiety symptoms during pregnancy [51]. Occupational studies have reported that altered urinary hormone levels (serotonin, norepinephrine, and dopamine) are associated with metal exposure, a finding that may explain the increased risk of anxiety [55].

#### 3.2.3. Cr Exposure and Health Outcomes in Childhood

Exposure to Cr may have detrimental effects not only when occurring during prenatal exposure but also in early life (Table 2). At this critical window of development, children are quite vulnerable to environmental chemical exposure due to an incomplete maturation of metabolic pathways and a higher magnitude of exposure per body weight.

In a cross-sectional study [56], a protective role of Cr against high blood pressure was observed. Compared to the lowest quartile of urinary Cr levels, children in the highest quartile had a 0.48-fold (95% CI: 0.25–0.88) decrease in the odds of hypertension. This association was more pronounced among 2–4-year-old children [56].

Exposure to chromium has been implicated in growth delay [57] and suppression of neurophysiological development in school-age children [58]. Exposed boys and girls in West Kazakhstan, aged 7–17 years, exhibited a retardation in annual height increase and body weight gain compared to children from a control area [57]. In children with short stature, an imbalance in hormonal status was observed based on a lack of activation of thyroid-stimulating hormones, as well as insufficient levels and an impaired ratio of gonadotropins [57]. Neuropsychological impairment was correlated with urinary and hair Cr levels in 393 children aged 6–11 years [58]. A 10-fold increase in urinary Cr was associated with a 5.99-point decrease in the full-scale IQ, an association manifested only in boys. In both sexes, high Cr concentrations were related to impaired selective attention and impulsivity [58]. Similarly to the placenta, Cr(VI) can cross the blood–brain barrier and cause oxidative stress due to its intracellular reduction. Consecutive cell apoptosis and hypoxia have been linked to cognitive impairment in animal models [58]. The role of chromium as a metallo-estrogen may also interfere with normal brain development [59].

Finally, in a longitudinal study on children residing in a mining area in Peru, hair Cr was associated with the presence of white lines on the nails. In this study, hair samples were taken in two time periods from a subset of children, and the comparison of Cr concentrations revealed a chronic exposure, as there was an increase from 0.26 μg/g in 2016 to 0.71 μg/g in 2018 [60].

**Table 2:** Summary of biomonitoring studies on environmental exposure to Cr in early life stages.

Study Characteristics (Area, Time, Population)	Study Design	Exposure Variables	Biomonitoring Data [(Mean ± SD) or Median]	Key Findings of Health Outcomes	Ref.
Prenatal exposure					
China, Hubei province 2012–2014 204 LBW cases 312 controls	Case control	Cr in maternal urine	Cr-U: 4.57 μg/g creatinine (range: 0.02–57.44 μg/g) (cases) Cr-U: 3.33 μg/g creatinine (range: 0.02–87.35 μg/g) (controls)	LBW risk OR = 1.77 (medium tertile), (95% CI: 0.95, 3.29) **OR = 2.48 (highest tertile), (95% CI: 1.33, 4.61)**	[36]
China, Wuhan 2014–2015 734 mother-infant pairs	Birth cohort	16 metals in umbilical cord serum (U, Cu, Pb, Se, Ba, Tl, Mn, Ni, Sr, As, Zn, Cd, V, Cr, Al, Co)	Cr-S: 10.4 μg/L (P25 6.45 μg/L, P75 16.3 μg/L)	Birth weight: no association Β = −0.02 (95% CI: −0.07, 0.03) per unit increase in lnCr	[37]
Israel 2016 975 mother-infant pairs	Cross-sectional	8 metals in maternal urine (As, Cd, Cr, Hg, Ni, Pb, Se, Tl)	Cr-U: 0.28 μg/g creatinine (P25 0.17 μg/g, P75 0.49 μg/g)	**Birth weight: β = −0.120 SD (95% CI: −0.202, −0.037)** **Birth length: β = −0.133 SD (95% CI: −0.215, −0.05)** per one-IQR increase in logCr-U	[38]
Canary Islands, Spain 2016 471 mothers	Cross-sectional	44 metals in cord blood (Ag, As, Au, Ba, Be, Bi, Cd, Ce, Cr, Cu, Dy, Eu, Er, Ga, Gd, Hg, Ho, In, La, Lu, Nb, Nd, Ni, Os, Pb, Pd, Pr, Pt, Ru, Sb, Se, Sm, Sn, Sr, Ta, Tb, Th, Tl, Tm, U, V, Y, Yb, Zn)	Cr-B: 1.10 ± 0.66 μg/L	Birth weight: no significant association When birth weight dichotomized at the P10, the sum of Cr, Ni, and Sb appeared as a risk factor for birth weight (**OR = 3.84; 95% CI = 1.42, 10.39**) in the multivariate analysis	[39]
Spain (5 counties) 2000–2008 327 mother–infant pairs	Birth cohort	6 metals in placenta (Cd, Pb, Mn, Cr, As, Hg)	Cr-placenta: 80.50 ng/g (P25 46.50 ng/g, P75 111.9 ng/g)	**Birth length: β = −0.68 cm, (95% CI: −1.33, −0.04)**, for Cr in the high vs. low tertile (>99.6 vs. <56.1 ng/g). **Gestational age: β = 0.56 weeks, 95% CI: 0.16, 0.97)**, for Cr in the middle vs. low tertile (56.1–99.6 vs. <56.1 ng/g)	[40]
China, Wuhan 2013–2016 3041 pregnant women	Birth cohort	Cr in maternal urine at 1st, 2nd, and 3rd trimesters	Cr-U: 0.98 μg/L (specific gravity-adjusted) (range: 0.61–1.75 μg/L)	1st trimester: **AC: −5.4% (95% CI: −9.6%, −1.2%),** **EFW: −5.6% (95% CI: −9.8%, −1.4%),** **ponderal index: −0.11 kg/m^3^ (95% CI: −0.19, −0.03)**, per unit increase in lnCr 2nd trimester: **AC: −7.0% (95% CI: −12.5%, −1.4%)** **EFW: −5.0% (95% CI: −10.6%, 0.6%)** **ponderal index: −0.15 kg/m^3^ (95% CI: −0.27, −0.03)** per unit increase in lnCr	[41]
China 2014–2017 1275 mother-infant pairs	Birth cohort	8 metals in maternal urine (Pb, Cd, Hg, As, Cr, V, Tl, Ba)	Cr-U: 0.75 μg/L (specific gravity-adjusted) (P25 0.43 μg/L, P75 1.24 μg/L)	**EFW: β = −0.06 (95% CI: −0.12, 0.00)** at 34–36 weeks of gestation Metal mixture: effect on EFW β_WQS_= −0.05 (95% CI: −0.09, −0.01), mainly driven by Cr (30.41%) at 34–36 weeks of gestation	[42]
China 2009–2013 147 SBP cases 381 controls	Nested case control	5 metals in maternal serum (As, Cd, Cr, Hg, Pb)	Cr-S: 0.275 μg/L (P25 0.196 μg/L, P75 0.417 μg/L) (no difference in cases and controls)	Spontaneous preterm birth (SBP): no association OR = 1.27 (95% CI: 0.84, 1.91)	[43]
China, Hubei 2012–2014 7290 pregnant women	Birth cohort	Cr in maternal urine	Cr-U: 1.86 μg/g creatinine (P25 0.86 μg/g, P75 5.65 μg/g) Cr-U: 1.01 μg/L (P25 0.61 μg/L, P75 2.09 μg/L)	**Gestational age (days): β = −0.68 (95% CI: −0.88, −0.48)** for continuous ln-Cr-U Risk for preterm birth: **OR = 1.55 (95% CI: 0.99, 2.42)** for the medium tertile vs low tertile of Cr-U **OR = 1.89 (95% CI: 1.13, 3.18)** for the high vs low tertile of Cr-U	[44]
China, Wuhan 2012–2014 5408 pregnant women of which 554 with PROM, 88 with preterm PROM	Birth cohort	Cr in maternal urine	Cr-U: 1.31 μg/g creatinine (P25 0.75 μg/g, P75 3.04 μg/g) (all) Cr-U: 2.39 μg/g creatinine (P25 1.13, P75 6.09) (PROM women) Cr-U: 4.37 μg/g creatinine (P25 1.72, P75 10.45) (preterm PROM women)	**OR = 1.47 (95% CI: 1.36, 1.58)** for one-unit increase in ln-Cr Risk for PROM: **OR = 1.42 (95% CI: 1.09, 1.84)** for the medium tertile; **OR = 2.77 (95% CI: 2.18, 3.52)** for the high vs low tertile of Cr-U Risk for preterm PROM: OR = 2.81 (95% CI: 0.92, 8.60) for the medium tertile vs low tertile of Cr-U **OR = 4.54 (95% CI: 1.58, 13.06)** for the high vs low tertile of Cr-U Higher associations in boys	[45]
Northern China 2003–2016 273 NTD cases 477 controls	Nested case control	10 metals in maternal blood (Cd, Co, Cr, Cu, Fe, Hg, Mn, Mo, Pb, Zn)	Cr-B: 1.06 μg/L (P25 0.85, P75 1.81) (cases) Cr-B: 1.01 μg/L (P25 0.80, P75 1.38) (controls)	Neural tube defects (NTDs): no association	[46]
China 2012–2013 112 infants with CHD 107 controls	Case control	6 metals in maternal blood (Pb, Cd, Cr, Cu, Hg, Se)	Cr-B: 3.63 μg/L (P25 2.09, P75 4.10) (cases) Cr-B: 3.57 μg/L (P25 3.27, P75 3.99) (controls) *p* = 0.160	Congenital heart defects (CHDs): no association OR = 0.24 (95% CI: 0.08, 1.69) for the middle tertile of Cr-B (3.40–3.79 μg/L) vs low tertile (<3.40 μg/L) OR = 0.84 (95% CI: 0.36, 1.96) for the high tertile of Cr-B (>3.79 μg/L) vs low tertile (<3.40 μg/L) (multivariable multi-element logistic regression model)	[47]
China 174 children with CS 85 controls	Case control	6 metals in child’s serum (Cr, Ni, Sn, As, Tl, Pb)	Cr-S: 2.10 μg/L (IQR range 18.10) (cases) Cr-S: 1.17 μg/L (IQR range: 0.53) (controls)	Craniosynostosis (CS): no association OR = 0.24 (95% CI: −0.59, 1.07) for Q2 vs Q1 OR = 0.81 (95% CI: −0.09, 1.72) for Q3 vs Q1 OR = 2.24 (95% CI: −0.13, 4.62) for Q4 vs Q1	[48]
China, Wuhan 2013–2016 628 mother-infant pairs	Birth cohort	7 metals in maternal urine at 1st, 2nd, 3rd trimesters (V, Cr, Ni, As, Cd, Tl, Pb)	Cr-U: 1.18 μg/g creatinine (P25 0.72 μg/g, P75 2.04 μg/g)	Allergic rhinitis in childhood: **OR = 1.41 (95% CI: 1.02, 1.95)** with Cr-U at 3rd trimester (logistic regression)/(+) effect in BKMR (+) trend with wheeze and eczema in BKMR	[49]
Palestine 2014 502 pregnant women 392 followed up in 6 months	Birth cohort	5 metals in maternal hair (Cr, Hg, V, Sr, U)	Cr-H: 0.97 ± 0.99 μg/g (range: 0.07–7.52 μg/g)	Child’s positive affectivity: **β = −0.13, *p* = 0.013** Not modified by maternal post-traumatic stress	[50]
During pregnancy					
USA, Boston/New York 2011 380 pregnant women	Cohort	6 metals in urine (Ba, Cd, Cr, Cs, Pb, Sb)	Cr-U: 0.62 μg/L (P25: 0.47 μg/L, P75: 0.83 μg/L)	**Anxiety score during pregnancy: 5.7% (95% CI: 23.9%, 95.7%) increase in the odds** of higher anxiety score, per one-quintile increase in the WQS index Top three contributing metals: Cd (61.8%), **Cr (14.7%)**, and Cs (12.7%)	[51]
China, Wuhan 2013–2016 305 GDM cases 305 controls	Nested case control	7 metals in plasma (Mg, Zn, Ca, Fe, Cu, Se, Cr)	Cr-P: 2.65 μg/L (P25: 1.26 μg/L, P75: 5.74 μg/L) (cases) Cr-P: 3.20 μg/L (P25: 1.53 μg/L, P75: 5.80 μg/L) (controls)	Gestational diabetes mellitus: no association OR = 0.93 (95% CI: 0.71, 1.22) per IQR increment	[53]
USA, Boston 2006–2008 28 preeclamptic 355 non-preeclamptic	Nested case control	17 metals in urine (As, Ba, Be, Cd, Cu, Cr, Hg, Mn, Mo, Ni, Pb, Se, Sn, Tl, U, W, Zn)	Cr-U: detected in 7 preeclamptic and in 50 non-preeclamptic	**Pre-eclampsia risk**: association with detection of Cr-U **HR = 3.48, (95% CI: 1.02, 11.8)** (limited number of observations)	[54]
During childhood					
China, Hubei 2019 1220 children aged 2–6 years old	Cross-sectional	23 metals in urine (Al, Ti, V, Cr, Mn, Fe, Co, Ni, Cu, Zn, As, Se, Rb, Sr, Mo, Cd, Sn, Sb, Ba, W, Tl, Pb, U)	Cr-U: 0.59 μg/L (P25 0.35 μg/L, P75 0.94 μg/L) (hypertensive) Cr-U: 0.71 μg/L (P25 0.42 μg/L, P75 1.68 μg/L) (normotensive)	**SBP: β = −0.96 (95% CI: −1.87, −0.04)** **DBP: β = −0.92 (95% CI: −1.83, −0.02)** Hypertension risk: β = 0.75 (95% CI: 0.55, 1.02)	[56]
West Kazakhstan 632 exposed children aged 7–17 years 621 unexposed children	Cross-sectional	5 metals in blood (Cr, Mn, Ni, Pb, Cu)	Cr-B: 1.8 μg/L ± 0.36 μg/L (exposed) Cr-B: 0.385 ± 0.18 μg/L (unexposed)	Short stature: OR = 3.578 imbalance of hormones in exposed children: decrease in T3, T4, TSH, gonadotropins (affects sexual development and puberty), STH, IGF-1 in the pre- and pubertal periods	[57]
Southern Spain 2010, 2012 393 children, aged 6–11	Cross-sectional	Cr in urine and hair	Cr-U: median 0.48 μg/L (0.96 μg/g creatinine), max: 21.0 μg/L (21.66 μg/g creatinine) Cr-H: median 0.32 μg/g, max 9.58 μg/g	For Cr-U Full-scale IQ: **β = −5.99 (95% CI: −11.9, −0.02)** in boys only Percentage of false alarms: **β = −0.05 (95% CI: −0.09, −0.01)** in boys Percentage of omissions **β = 0.03 (95% CI: 0.00, 0.05)** in boys Latency in reaction time test: **β = 36.90 (95% CI: 3.50, 70.30)** in girls Latency in reaction time test: β = 68.35 (95% CI: 6.60, 130.12) in boys For Cr-H Response latency in selective attention test: **β = −55.01 (95% CI: −74.04, −35.96)** in both sexes	[58]
Peru 2016, 2018 78 exposed children, average 10 years old 16 unexposed	Longitudinal	21 metals in hair (Al, Sb, As, B, Ba, Be, Cd, Co, Cr, Fe, Mn, Hg, Mo, Ni, Pb, Cu, Se, Sn, Tl, V, Zn)	Cr-H: 0.83 μg/g in roots, 1.61 μg/g in tips (exposed) Cr-H: 0.39 μg/g in roots, 0.88 μg/g in tips (unexposed)	**White lines on nails**: χ^2^ test, *p* = 0.002	[60]

(+): positive association, (−): negative association, AC: abdominal circumference, BKMR: Bayesian kernel machine regression, CHDs: congenital heart defects, CI: confidence interval, Cr-B: Cr in blood, Cr-H: Cr in hair, Cr-P: Cr in plasma, Cr-S: Cr in serum, Cr-U: Cr in urine, CS: craniosynostosis, DBP: diastolic blood pressure, EFW: estimated fetal weight, GDM: gestational diabetes mellitus, HR: hazard ratio, IGF: insulin-like growth factor, IQ: intelligence quotient, IQR: interquartile range, LBW: low birth weight, NTDs: neural tube defects, OR: odds ratio, P10: 10th percentile, P25: 25th percentile, P75: 75th percentile, PROM: premature rupture of membranes, SBP: systolic blood pressure, SPB: spontaneous preterm birth, STH: somatotropic hormone, T3: triiodothyronine, T4: thyroxine, TSH: thyroid-stimulating hormone, WQS: weighted quantile sum. Significant associations are indicated in bold.

Studies that examined exposure to metal mixtures employed either classical linear or logistic regression models, in which levels of other metals were included as additional covariates [39,40,47,48,50], or advanced statistical methods, e.g., weighted quantile sum regression (WQSR), to estimate the relationship between metal mixtures and health effects [37,42], or Bayesian kernel machine regression (BKMR) models [38] or even a combination of traditional and advanced techniques [46,49]. Furthermore, in the majority of the studies, sensitivity analysis was performed [37,40,41,42,44,45,46,47,49] to assess the robustness of the findings. 

#### 3.2.4. Cr Exposure and Health Effects during Adulthood

Table 3 presents the studies of health outcomes during adulthood being associated with Cr exposure.

- Chromium and hypertension

Hypertension and blood pressure in relation to internal Cr levels were examined in four studies [29,61,62,63]. No significant association was revealed between Cr in blood [29,61], Cr in urine [61], Cr in hair [29], or Cr in toenails [63] and hypertension [61], pre-hypertension [61], or blood pressure [29,63]. Notably, the Chinese cross-sectional study [61] recruited a large sample size (11,037 adults), of which 34.9% were classified as pre-hypertensive and 36.1% as hypertensive, and the associations of single metals or their mixture were assessed through advanced statistical methods after controlling for demographic, lifestyle behavior, and dietary intake data, while sensitivity analysis confirmed their findings. In the Greek environmental epidemiological study [29], however, despite the lack of association of blood pressure with internal Cr levels, a slight positive association was observed, after controlling for various covariates, between lifetime Cr exposure dose and systolic and diastolic blood pressure in 304 adults, of which 38.7% were classified as hypertensive [29]. In contrast, a slight negative correlation of systolic blood pressure with urinary Cr levels was found in a case control study that enrolled 69 patients with coronary heart disease (CHD) and 147 healthy adults in China [62]. In this population, systolic blood pressure in patients with CHD (mean: 132.4 ± 16.7 mm Hg) was significantly higher than in healthy adults (mean: 125.8 ± 18.5 mm Hg) and the negative correlation was not adjusted for potential confounders [62].

- Chromium and cardiovascular system

Only two studies were extracted in which the relationship of cardiac function-related health outcomes to Cr exposure was investigated. The first was the Chinese case control study reported previously [62]. Its primary objective was to explore associations between metal exposure and coronary heart disease (CHD) risk. Even though significantly higher Cr levels were found in healthy adults compared to CHD patients, the final logistic regression model showed no association between urinary Cr and coronary heart disease after adjustment for age, sex, and smoking status.

The association of heart rate variability (HRV) and urinary concentrations of metals was examined in a cross-sectional study of 2004 adults in Wuhan, China [64]. HRV is a physical indicator of cardiac autonomic balance and reflects autonomic regulation of rhythmic heart rate. No association was observed between urine Cr levels and HRV.

- Cr and hematological/biochemical parameters (liver function and oxidative stress)

The historically polluted area of Jinzhou City in Liaoning province in China, which has attracted attention due to conflicting findings of estimated cancer mortality rates [22,23,24,25], has served as a study area in three biomonitoring studies [65,66,67] investigating potential differences in hematological and biochemical parameters between exposed and unexposed people. Other related studies were conducted in other parts of China [68,69,70], Pakistan [71], India [28,72], South Korea [63], Spain [73], Greece [29], and the USA [74,75]. 

Results regarding the association of hematological parameters with Cr exposure dose [28,29] or blood Cr [67] are contradictory. Higher red blood cell (RBC) counts were reported in an environmentally exposed Indian population [28], while no association of RBC with blood Cr levels was found in the exposed Chinese and Greek populations [29,67]. It has been reported that exposure of red blood cells to Cr(VI) can lead to eryptosis, which aims to prevent hemolysis of defective erythrocytes, although it may lead to anemia [76]. A strong positive association with hemoglobin was observed in [67], while a weak negative one was found in [29]. Finally, two cross-sectional studies of environmental Cr exposure reported a decrease in platelet counts with increasing Cr exposure [28] or hair Cr levels [29]. These discrepancies may be attributed to the different levels of exposure experienced by the populations studied, as well as to the cross-sectional nature of the studies.

As for the main biochemical parameters and their relationship with Cr levels in the body, 14 biomonitoring studies were identified (Table 3). Total protein and albumin were found to be positively associated with Cr exposure dose and Cr in blood and hair in the Greek study [29], while a negative association was found by linear regression in the Chinese population [65]; however, the non-linear exposure–response function generated by the Bayesian kernel machine regression models in [65] showed that when Cr was at low levels, its relationship with both albumin and total protein was towards the positive direction. 

The other common parameters of liver function are aminotransferases; their increase manifests hepatic cell damage. Contradictory results have been reported. Serum AST was positively associated with urinary Cr in one Chinese study [62] and negatively associated with serum Cr in another Chinese study [68], while no significant association was observed with both AST and ALT and internal levels of Cr in two other studies [29,65]. 

Regarding markers of lipid metabolism, positive associations of total triglycerides [29], total cholesterol [29,62,66], and LDL [29,66] and a negative relationship of HDL [29,66] with Cr concentrations were observed; however, another study showed a protective association of Cr with the risk of dyslipidemia [69], whereas in two other studies, no significant associations were observed between Cr in blood [67] or Cr in toenails [63] with total cholesterol [63,67], LDL [67], and HDL [63]. These inconsistent results mainly concern cross-sectional studies, which reveal only associations, do not allow causal inferences, and were conducted in populations living in different geographic areas with non-comparable exposure levels and with wide variation in Cr levels in the examined biomarkers. Nevertheless, in the Chinese study [69], in which a significant proportion of the study population was followed up with for 3 years, the beneficial effect of Cr on decreasing total triglycerides and increasing HDL, which was observed at baseline, lost its statistical significance at follow-up. Another discrepancy comes from the fact that some studies [65,66,68] examined the cumulative effect of exposure to multiple metals on health outcomes via Bayesian kernel machine regression, which not only estimates the overall effect of the metal mixture but also the contribution of a single metal on the overall effect by identifying potential non-linear, non-additive associations between metals and outcomes. Even though this analysis better represents the actual exposure occurring in populations, many of the observed associations may be the result of either synergistic or competitive interactions of Cr with other metals [68]. Moreover, in some cases, the relationship of Cr with a health outcome explained by traditional regression models appears to change direction when examined by BKMR models e.g., in [65]. The latter can also identify a positive (or negative) association below a certain Cr concentration and a negative (or positive) relationship above this concentration, presenting a U-shape or inverted U-shape, which better reflects the actual interaction of metals with biological effects.

A recurring pattern in the relevant studies concerns the association of internal Cr levels with markers of oxidative stress [27,67,73]. It appears that oxidative stress markers (CAT, GSH-Px, GSSG/GSH ratio) and the end-products of lipid peroxidation (MDA) and DNA oxidation (8-OHdG) present a continuous positive association with Cr internal levels, all indicating an activation of the antioxidant system, while the decrease in SOD activity [67], found also in study [27], may be a sign of the impairment of the antioxidant system, according to the researchers.

Finally, the relationship of diabetes risk with Cr intake was investigated in three studies [72,74,75]. A protective role of dietary Cr supplementation [74] or detectable urinary Cr levels [75] against diabetes was revealed in the two USA cohorts, involving a total of 29,776 people. Conversely, significantly higher odds of type II diabetes were observed in people in the highest quartile of urinary Cr levels in a cross-sectional study in rural India [72]. Furthermore, no association was observed between internal Cr concentrations and fasting glucose levels in two cross-sectional studies, one involving elderly people in China [70] and a second Korean adults [63], while a negative unadjusted correlation of fasting glucose and glycated HbA1c was reported in [62].

- Cr and obesity

The relationship of BMI and obesity with urinary Cr levels was investigated in two studies [77,78]. The first one was conducted in the exposed population residing in the polluted area of Jinzhou city, and urinary levels of four metals (Cr, Cd, Pb, and Mn) were determined. Urinary Cr did not differ among the three groups of weight classification (overweight, normal, underweight) (Wilcoxon test, *p* = 0.354). The analysis showed that urinary Cr concentration exhibited a nonlinear relationship with BMI, with a positive association at levels below the median of urinary Cr (3.48 μg/L) and a negative association above the median [77]. Tinkov et al. [78] did not find an association between urinary Cr levels and BMI values in 199 lean and 196 obese adults. However, they did find an inverse association of serum Cr levels with BMI (β = −0.320, *p* < 0.001), after adjusting for age and sex, in a linear model that explained about 30% of the BMI variability. According to the researchers, Cr may counteract insulin resistance, leading to 47% lower serum Cr in obese subjects. Levels of Cr in hair were significantly higher in obese than in lean subjects, even though the final model did not find an association between hair Cr levels and BMI values (β = 0.085, *p* = 0.117), after adjusting for age and sex.

- Cr and renal system

Chronic kidney disease (CKD) is another health problem that has been studied in relation to Cr exposure [79,80,81,82]. A strong negative association between urinary Cr levels and estimated glomerular filtration rate (eGFR), which is derived through linear regression models, has been reported in a population with normal ranges of eGFR values [80]. A nonlinear dose–response relationship was observed between urinary Cr in men with a prevalence of nephrolithiasis in a Chinese cross-sectional study [83]. When the urinary Cr concentration was in the range of 17.78 to 25.12 μg/L, the risk of nephrolithiasis was significantly increased by 24%. However, this study population exhibited quite high urinary Cr concentrations compared to other studies.

In contrast, higher odds of developing CKD, defined as eGFR < 60 mL/min/1.73 m^2^, were not evident in two other cross-sectional studies, in which subjects with impaired renal function comprised 2.5% [82] and 14% [79] of the total study population. Similarly, no higher hazard ratio for end-stage renal disease was observed in response to Cr concentration in residential soil [81]. No association of urinary Cr levels and immunoglobulin A nephropathy (IgAN), which is the most common type of glomerulonephritis in adults worldwide, was found in a Chinese case control study of 160 IgAN patients and 480 healthy controls [84].

Possible mechanisms involved in impaired renal function have been suggested to include the reduction of Cr(VI) to Cr(III) in the stomach and gastrointestinal tract, leading to an increase in renal Cr excretion. In addition, simultaneous exposure to Cr, Pb, and Cd might cause a further decrease in glomerular filtration rate, possibly mediated by oxidative stress in the kidney and thus increasing the risk of renal damage [79,80,83].

**Table 3:** Summary of the studies on health outcomes during adulthood being associated with levels of Cr biomarkers.

Study Characteristics (Area, Time, Population)	Study Design	Exposure Variables	Biomonitoring Data [(Mean± SD) or Median]	Key Findings on Health Outcomes		Ref.
Hypertension						
China, 31 provinces 2017–2018 11,037 adults	Cross-sectional	13 metals in blood and urine (Sb, As, Cd, Pb, Hg, Tl, Cr, Co, Mn, Mo, Ni, Se, Sn)	Cr-B = 0.42 μg/L (P25: 0.16 μg/L, P75: 0.81 μg/L) Cr-U = 0.57 μg/L (P25: 0.28 μg/L, P75: 1.12 μg/L)	No association with hypertension, pre-hypertension, or blood pressure Hypertension: OR = 0.90 (95% CI: 0.63, 1.29) for Cr-B high vs low quartile, OR = 0.98 (95% CI: 0.75, 1.28) for Cr-U high vs low quartile. Pre-hypertension: OR = 0.87 (95% CI: 0.66, 1.17) for Cr-B high vs low quartile, OR = 0.94 (95% CI: 0.74, 1.19) for Cr-U high vs low quartile.		[61]
Cardiovascular disease						
China, Guangzhou 2021 69 CHD patients 147 controls	Case control	10 metals in urine (Cr, Fe, Co, Ni, Cu, As, Se, Cd, Sn, Hg)	Cr-U = 0.50 μg/L (P25: 0.34 μg/L, P75: 0.74 μg/L) (patients) Cr-U = 0.80 μg/L (P25: 0.58 μg/L, P75: 1.18 μg/L) (controls)	CHD: OR= 0.431 (95% CI: 0.151, 1.229) **SBP: Spearman’s r = −0.192, *p* = 0.017 ** **Glycated HbA1c: Spearman’s r = −0.241, *p* = 0.001 ** **Fasting blood-glucose: Spearman’s r = −0.190, *p* = 0.022 ** **HDL: Spearman’s r = 0.149, *p* = 0.032 ** **Total Cholesterol: Spearman’s r = 0.150, *p* = 0.031** **AST: Spearman’s r = 0.164, *p* = 0.032**		[62]
China, Wuhan 2011 2004 adults, aged 18–80	Cross-sectional	23 metals in urine (Al, Ti, V, Cr, Mn, Fe, Co, Ni, Cu, Zn, As, Se, Rb, Sr, Mo, Cd, Sn, Sb, Ba, W, Tl, Pb, U)	Cr-U: 1.46 μg/L (P25: 0.93 μg/L, P75: 2.24 μg/L)	No association with heart rate variability (estimators not mentioned)		[64]
Hematological/biochemical						
Greece, Voiotia prefecture 2012–2014 122 currently exposed 115 exposed in past 67 non-exposed	Cross-sectional retrospective	Cr in blood Cr in hair	Cr-B 0.32 μg/L (range: <0.18–0.92 μg/L) (no difference) Cr-H: 0.31 μg/g (P25: 0.21 μg/g, P75: 0.46 μg/g) (currently exposed) Cr-H: 0.12 μg/g (P25: 0.07 μg/g, P75: 0.29 μg/g) (exposed in past) Cr-H: 0.22 μg/g (P25: 0.13 μg/g, P75: 0.32 μg/g) (non-exposed)	**Signif. associations with Cr-B** **Glu(ln): β = 0.248, *p* < 0.001** **Urea: β = −0.143, *p* = 0.012** **Potassium: β = 0.120, *p* = 0.038** **Alkaline phosphatase: β = 0.216, *p* < 0.001** **γ-GT: β = 0.109, *p* = 0.041** **LDH: β = 0.123, *p* = 0.033** **Amylase: β = 0.177, *p* = 0.002 ** **Albumin: β = 0.232, *p* < 0.001** **TP: β = 0.237, *p* < 0.001** **Calcium: β = 0.219, *p* < 0.001**	**Signif. associations with Cr-H(ln)** **Hematocrit: β = −0.099, *p* = 0.041** **PLT: β = −0.202, *p* < 0.001** **Platelecrit: β = −0.229, *p* < 0.001** **MCHC: β = −0.191, *p* = 0.001** **RBC-DW: β = −0.191, *p* = 0.001** **Glu(ln): β = 0.193, *p* = 0.001** **TC: β = 0.268, *p* < 0.001** **LDL: β = 0.216, *p* < 0.001** **Alkaline phosphatase: β = 0.131, *p* = 0.026** **LDH: β = 0.175, *p* = 0.003** **Amylase: β = 0.262, *p* < 0.001** **Albumin: β = 0.164, *p* = 0.005** **TP: β = 0.376, *p* < 0.001** **Calcium: β = 0.321, *p* < 0.001** **Phosphate: β = 0.121, *p* = 0.041** **Potassium: β = 0.127, *p* = 0.033** **Sodium: β = −0.142, *p* = 0.018**	[29]
China, Liaoning Province, Jinzhou City 2016–2018 1171 adults: 364 exposed, 807 non-exposed	Cross-sectional	4 metals in urine (Cr, Cd, Pb, Mn)	Cr-U: 4.67 μg/L (P25: 3.05 μg/L, P75: 6.01 μg/L) (exposed) Cr-U: 4.22 μg/L (P25: 1.74 μg/L, P75: 5.55 μg/L) (non-exposed)	**Total protein: β = −0.57 (95% CI: −0.89, −0.26)** with GLM/ PIP for Cr = 0.91 with BKMR **ALB: β = −0.27, (95% CI: −0.47, −0.07)**/PIP for Cr = 1.00 with BKMR ALT: β = 0.63 (95% CI: −0.02, 1.28) with GLM / weight of Cr = 0.38 with quantile g-computation AST: β = 0.24 (95% CI: −0.22, 0.71) with GLM / weight of Cr = −0.83 with quantile g-computation		[65]
China, Liaoning province, Jinzhou city 2017–2019 1121 older adults (mean: 62.4 ± 10.6 years) 433 exposed 688 non-exposed	Cross-sectional	4 metals in urine (Cr, Cd, Pb, Mn)	Cr-U: 4.02 μg/L (P25: 2.64 μg/L, P75: 5.28 μg/L) (exposed) Cr-U: 3.88 μg/L (P25: 2.74 μg/L, P75: 5.17 μg/L) (non-exposed)	HDL: β = 0.001 (95% CI: −0.03, 0.02) with GLM/**(−) association with BKMR** LDL: β = 0.02 (95% CI: −0.01, 0.04) with GLM/**(+) association with BKMR** Triglycerides: β = 0.01 (95% CI: −0.01, 0.03) with GLM Total Cholesterol: β = 0.01 (95% CI: −0.02, 0.03)/(+) association with BKMR		[66]
China, Liaoning province, Jinzhou city 2016 585 adults: 282 exposed 303 non-exposed	Cross-sectional	Cr, Pb, Mn in blood	Cr-B= 0.92 μg/L (P25: 0.83 μg/L, P75: 1.02 μg/L) (exposed) Cr-B= 0.88 μg/L (P25: 0.80 μg/L, P75: 0.99 μg/L) (non-exposed)	RBC: β = 0.16 (95% CI: −0.0004, 0.33) **Hb: β = 8.52 (95% CI: 3.10, 13.95)** TC: β = −0.08 (95% CI: −0.45, 0.29) LDL: β = −0.08 (95% CI: −0.38, 0.22) **SOD: (−) exposure–response relationship, *p* for trend = 0.017**		[67]
China, rural northwest 2018–2019 785 adults	Cross-sectional	Cr, Co, Cd, Pb in serum	Cr-S: 2.05 μg/L (P25: 0.68 μg/L, P75: 4.98 μg/L)	**AST: β = −0.099 (95% CI: −0.035, −0.003)** **ALT: β = −0.070 (95% CI: −0.043, 0.000)** (single-metal model)		[68]
China, Wuhan city and Zhuhai city 2012–2015 3762 adults, 18–80 years old (in 2012, for cross-sectional) Follow-up: 1750 adults for 3 years	Cohort and cross-sectional	Cr in urine	Cr-U: 1.60 µg/L (0.13 μg/mmol creatinine, P25: 0.08, P75: 0.21)	**TG: β = −0.25 mmol/L (95% CI: −0.38, −0.11)** per 1-unit increase of logCr-U TC: β = −0.05 (95% CI: −0.19, 0.09) per one-unit increase of logCr-U LDL: β = −0.06 (95% CI: −0.16, 0.04) per one-unit increase of logCr-U **HDL: β = 0.05 (95% CI: 0.005, 0.10)** per one-unit increase of logCr-U		[69]
China, Beijing 2016 275 adults, 68.9 median age	Cross-sectional	15 metals in urine (Al, Cr, Mn, Fe, Co, Ni, Cu, Zn, As, Se, Sr, Cd, Cs, Ba, Pb).	Cr-U: 0.268 μg/g creatinine (P25: 0.189 μg/g, P75: 0.381 μg/g)	Fasting plasma Glu: β = 0.024 (95% CI: −0.033, 0.081) with GLM Fasting plasma Glu: no association with BKMR		[70]
Pakistan, 4 areas (control, low, medium, high risk of exposure) 48 children and adults: 12 from each area	Cross-sectional	9 metals in blood, urine, hair, nails (Cd, Cr, Pb, Cu, Ni, Co, Mn, Fe, Zn)	Cr-B: mean values: 0.07 (control area)–0.27 (medium risk) Cr-U: mean values: 0.18 (control area)–0.62 (low risk) Cr-H mean values: 0.25 (control area)–0.59 (medium risk) Cr-Nails mean values: 0.34 (control area)–0.56 (medium risk)	**GSH-Px: Spearman’s r = −0.315, *p* = 0.035**		[71]
India, rural south 2015 847 adults, 715 possibly exposed	Cross-sectional	8 metals in urine (Cd, As, Pb, Cr, Al, Zn, Cu, Ni)		Diabetes: OD = 1.05 (95% CI: 0.58, 2.02) for Q2 vs. Q1 OD = 1.87 (95% CI: 0.99, 3.51) for Q3 vs. Q1 **OD = 2.40 (95% CI: 1.26, 4.56)** for Q4 vs. Q1 **Glycated HbA_1c_: Spearman’s r = 0.12, *p* < 0.01** No correlation with SBP, DBP, BMI, TC, LDL (Spearman’s r)		[72]
South Korea 2012–2013 500 adults, aged >35	Cross-sectional		Cr-Toenail: range 0.003–5.76 μg/g	Fasting Glu, SPB, DBP, TG, HDL, waist circumference: No association (*p*_trend_ > 0.050)		[55]
Spain 2013 1440 adults	Cross-sectional	9 metals in urine (Sb, Ba, Cd, Cr, Co, Cu, Mo, V, Zn)	Cr-U= 3.5 μg/g (P25: 2.2 μg/g, P75: 5.8 μg/g)	GSSG/GSH: **Geometric mean ratios = 1.23 (95% CI: 1.04, 1.46)** (P80 vs. P20)/non-linear (+) relation of Cr with GSSG/GSH with BKMR MDA and 8-OHdG: No association with Cr-U		[73]
USA 1999–2010 28,539 adults	Cohort	Consumption of Cr-dietary supplement		Risk of diabetes and/or HbA1c ≥ 6.5% **OR = 0.81 (95% CI: 0.71, 0.91)** for Cr supplement use **OR = 0.83 (95% CI: 0.72, 0.96)** for ≥ 2000 mg Cr/30 d than no supplement **OR = 0.78 (95% CI: 0.66, 0.92)** for < 2000 mg Cr/30 d than no supplement		[74]
USA 1999–2016 1237 women, aged 45–56	Cohort	20 metals in urine (As, Ba, Be, Cd, Co, Cr, Cs, Cu, Hg, Mn, Mo, Ni, Pb, Sb, Sn, Tl, U, V, W, Zn)	Cr-U median < 0.4 μg/L Cr > 0.4 μg/L in 24.3% of the population	Incident diabetes: **HR = 0.71 (95% CI: 0.50, 1.01)** for those with Cr > LOD compared to those with Cr < LOD		[75]
Obesity						
China, Liaoning province, Jinzhou City 2017–2019 1187 adults: 349 exposed 838 non-exposed	Cross-sectional	4 metals in urine (Cr, Cd, Pb, Mn)	Cr-U: 3.48 μg/L (P25: 1.84 μg/L, P75: 4.93 μg/L)	BMI: ***p*_trend_ = 0.023** (linear regression with quartiles of Cr-U) Positive association below Cr-U median and negative above Cr-U median (with restricted cubic spline) Waist circumference ***p*_trend_ = 0.018** (linear regression with quartiles of Cr-U)		[77]
Russia 395 adults 20–60 years old 199 lean 196 obese	Cross-sectional	4 metals in serum, hair and urine (Se, Zn, Cr, V)	Cr-S: 1.664 ng/mL (P25: 1.069, P75: 2.187) (normal weighted) Cr-S: 0.890 ng/mL (P25: 0.618, P75: 1.383) (obese) Cr-H: 0.065 μg/g (P25: 0.037, P75: 0.116) (normal weighted) Cr-H: 0.098 μg/g (P25: 0.059, P75: 0.199) (obese) Cr-U: 0.610 ng/mL (P25: 0.345, P75: 0.964) (normal weighted) Cr-U: 0.670 ng/mL (P25: 0.377, P75: 1.728) (obese)	BMI **Cr-S: β = −0.320, *p* < 0.001** Cr-U: β = −0.054, *p* = 0.328 Cr-H: β = 0.085, *p* = 0.117		[78]
Renal function						
Taiwan 2011 1643 adults, >50 years old: 1418 healthy, 225 with impaired eGFR	Cross-sectional	5 metals in blood (As, Cd, Pb, Ni, Cr)	Cr-B: 0.15 μg/L (IQR: 2.25)	Risk for eGFR < 60 mL/min/1.73 m^2^ OR= 1.01 (95% CI: 0.97, 1.06)		[79]
Taiwan 2005–2008 360 healthy adults, 19–84 years old	Cross-sectional	Cr, Pb, Cd in urine	Cr-U (geometric mean): 0.83 μg/L (95% CI 0.76 μg/L, 0.92 μg/L)	eGFR: **β = −5.99 (95% CI: −9.70, −2.27)** with logCr-U [mean eGFR: 100.3 mL/min/1.73 m^2^ (SD: 20.42) in men, 105.9 mL/min/1.73 m^2^ (SD: 20.26) in women]		[80]
Taiwan, Changhua County, 2003–2015 2343 CKD patients, 20–90 years old: 533 with progression to end-stage renal disease [ESRD] and 1810 non-ESRD	Cohort of patients	8 metals in soil (As, Cd, Cr, Hg, Cu, Pb, Ni, Zn)	Cr-Soil: 6.30 ± 11.04 mg/kg (for ESRD patients) Cr-Soil: 5.55 ± 8.89 mg/kg (for non-ESRD patients)	ESDR: HR = 1.072 (95% CI: 0.998, 1.152) for logCr in residential soil [mean eGFRs 17.8 ± 12.3 mL/min per 1.73 m^2^ (in ESRD patients) vs. 33.7 ± 20.0 (in non-ESRD patients)]		[81]
China, rural areas 2016–2017 3553 adults	Cross-sectional	23 metals in plasma and urine (Al, Ti, V, Cr, Mn, Fe, Co, Ni, Cu, Zn, As, Se, Rb, Sr, Mo, Cd, Sn, Sb, Ba, W, Tl, Pb, U)	Cr in plasma: app. 10 μg/L Cr-U: app. 2 μg/L	No association with abnormal eGFR (Wilcoxon rank sum test, *p* > 0.050) abnormal eGFR < 60 mL/min/1.73 m^2^: 2.5% of the population		[82]
China 2018 1502 men, aged 30–79, of which 357 with nephrolithiasis	Cross-sectional	5 metals in urine (As, Pb, Cd, Hg, Cr)	Cr-U: 30.20 µg/L (P25: 16.57 μg/L, P75: 45.12 μg/L)	Nephrolithiasis risk: when Cr-U from 17.78 to 25.12 μg/L **OR = 1.24 (95% CI: 1.06, 1.45)** [eGFR (mL/min/1.73 m^2^): 73.31 ± 16.64 (in non-nephrolithiasis subjects) vs. 68.97 ± 17.59 (nephrolithiasis subjects)]		[83]
China 2016–2021 160 IgAN patients 480 healthy controls	Case control	8 metals in plasma (V, Cr, Mn, Co, Cu, Zn, As, Pb)	Cr-U: 1.88 µg/L (P25: 0.95 μg/L, P75: 3.74 μg/L) (patients) Cr-U: 2.63 µg/L (P25: 2.27 μg/L, P75: 3.02 μg/L) (controls) Cr-U lower in patients (*p* < 0.001)	IgAN risk OR = 0.722 (95% CI: 0.456, 1.14) [eGFR (mL/min/1.73 m^2^): 71.1 ± 46.2 (cases) vs 104.0 ± 22.2 (controls]		[84]
Other health outcomes						
USA, 16 counties 2005–2009 413 reproductive-aged men	Cross-sectional	20 metals in urine (Co, Cr, Cu, Mn, Mo, Ni, Se, Zn, Sb, As, Ba, Be, Cd, Pb, Pt, Te, Tl, Sn, W, U).	Cr-U: 0.62 μg/L (IQR: 0.66)	**Total sperm count: β = 1.87, *p* = 0.003** **DNA fragmentation index β = −5.08, *p* = 0.0009** [91.3% of the samples within normal range for total sperm count]		[85]
China 2017–2019 210 age-related cataract patients 210 controls	Case control	14 metals in urine (As, Ba, Cr, Co, Cu, Pb, Li, Mg, Mn, Mo, Fe, Se, Sr, Zn)	Cr-U: 25.02 μg/L (P25: 10.61 μg/L, P75: 36.25 μg/L)	Age-related cataract: **OR = 3.71, (95% CI: 1.52, 9.08)** (Q4 vs. Q1, in single model) No association in multi-element model		[86]
Spain 2007–2016 458 affected municipalities	Ecological mortality	Distance 20 km or less from emission points 156 Cr-exposed municipalities		Motor neuron disease **SMR = 1.12 (95% CI: 1.08, 1.17) for exposed municipalities** 15.5% higher risk of MND		[87]

(+): positive association, (−): negative association, ALT: alanine transaminase, AST: aspartate aminotransferase, BKMR: Bayesian kernel machine regression, BMI: body mass index, CAT: catalase, CHD: coronary heart disease, CI: confidence interval, CKD: chromic kidney disease, Cr-B: Cr in blood. Cr-H: Cr in hair, Cr-U: Cr in urine, Cr-S: Cr in serum, DPB: diastolic blood pressure, eGFR: estimated glomerular filtration rate, ESRD: end-stage renal disease, Hb: hemoglobin, HDL: high-density lipoprotein, HR: hazard ratio, γ-GT: γ-glutamyl transpeptidase, Glu: glucose, glycated HbA1c: glycated hemoglobin, GLMs: generalized linear models, GSH-Px: glutathione peroxidase, GSSG/GSH: oxidized glutathione/reduced glutathione ratio, IgAN: immunoglobulin A nephropathy, LDH: lactate dehydrogenase, LDL: low-density lipoprotein, LOD: limit of detection, MCHC: mean cell hemoglobin concentration, MDA: malondialdehyde, OR: odds ratio, P25: 25th percentile, P75: 75th percentile, PIP: posterior inclusion probability, PLT: platelet count, Q: quartile, RBC: red blood cell count, RBC-DW: red blood cell distribution width, SD: standard deviation, SMR: standardized mortality rate, SOD: superoxide dismutase, SPB: systolic blood pressure, TC: total cholesterol, TGs: total triglycerides, TPs: total proteins, 8-OHdG: 8-hydroxy-2′-deoxyguanosine. Significant associations are indicated in bold.

- Cr and other health outcomes

One study [85] examined associations between urinary metal concentrations and seven measures of semen quality in a subset of the US LIFE cohort study. A beneficial association of urinary Cr with total sperm count and a negative, beneficial association with DNA fragmentation index were found, perhaps due to the antioxidant activity of Cr. However, the researchers urge caution in interpreting their findings, at least until they are verified in future studies. 

Urinary Cr levels were positively associated with age-related cataract (ARC) in a Chinese case control study [86]. Cataract is the leading cause among many blind eye diseases in the world. However, the multi-element model excluded Cr from the analysis as a relatively unimportant variable. 

Mortality rates due to motor neuron disease (MND) were examined in relation to metal concentrations in river basins in an ecological mortality study [87]. Motor neuron disease (MND) is a neurodegenerative, currently incurable condition of unknown etiology in which motor neuron functions progressively decline in the central nervous system, leading to death. Areas exposed to seven metals, including Cr, were identified through the European Pollutant Release and Transfer Register (E-PRTR) database after considering a 20 km river section downstream from the emission point of the metals. Significant SMR differences were observed in exposed versus reference areas. People residing in Cr-exposed areas had a 15.7% higher risk of MND.

- Cr and cancers

Chromium has been examined as a biomarker of exposure in various types of cancers (Table 4). Breast cancer was explored in relation to Cr, among other metals, in urine [88] and serum [89] in two case control studies. In both studies, Cr levels were similar between breast cancer cases and controls, and in no study was a significant association obtained. Notably, the metal mixture of Cr, Ni, Sb, Al, Pb, and Sn, which was extracted via principal component analysis, was associated with 1.15 times (95% CI: 1.06–1.25) higher odds of breast cancer risk [88].

Concerning skin cancer, in a large prospective study [90], Cr concentrations in toenails were associated with higher risk of incident basal cell carcinoma (BCC) and squamous cell carcinoma (SCC) in women, although the latter was not statistically significant. Toenail Cr levels were not associated with melanoma risk in either women or men. Even if these results provide some indication, the authors note the lack of biological explanations for the gender difference and ask for further studies for confirmation. 

In a case control study on urothelial carcinoma [91], even if five metals were determined in urine, only Cr differed according to the primary sites of cancer and increased in the following order: bladder tumors (mean ± SE: 1.94 ± 0.35 μg/g creatinine) < upper urinary tract tumors (mean ± SE: 2.22 ± 0.79 μg/g creatinine) < combined tumors (mean ± SE: 5.80 ± 2.43 μg/g creatinine). No difference was observed between urinary Cr levels and indices of cancer severity, such as stage, grade, or muscle invasive. Adjusted ORs of urothelial cancer risk were 2.01 (95% CI: 1.12–3.60) for those in the second tertile (Cr levels 0.18–0.45 μg/g creatinine) and 5.78 (95% CI: 3.37–9.90) for those in the upper tertile (Cr levels ≥ 0.45 μg/g creatinine).

Metal urine concentrations were evaluated in relation to the aggressive clinicopathologic characteristics of papillary thyroid carcinoma (PTC) in a patient study [92]. Only the risk of multifocality was significantly associated with urine Cr quartiles [Q4 vs. Q1, OR = 1.68, 95% CI: 1.02–2.77] after adjustment for confounders. Multifocality was defined as two or more tumor foci in the thyroid gland. In the multi-microelements model, which assessed the simultaneous effects of co-exposure to multiple microelements, urinary Cr levels did not present any association.

In a clinic-based, case control study of the Mayo Clinic [93], risk of pancreatic cancer was examined in regard to self-reported exposures to chemicals and heavy metals. Data were collected from 2000 to 2014, and 2092 patients diagnosed with pancreatic ductal adenocarcinoma and 2353 matched controls participated. A suggestive, although not statistically significant, association was found between affirmation of regular exposure to chromium and pancreatic cancer with an OR of 1.42 (95% CI: 0.89–2.26).

Significantly lower Cr levels were found in cancerous tissues excised from 49 bladder cancer patients compared to 36 tissues from controls who underwent bladder resection [94]. The finding was attributed to a dilution effect of Cr in the tumor due to its rapid proliferation, according to the authors. 

Finally, in a Spanish ecological study that examined cancer deaths during 1999–2008 in the whole country [95], Cr topsoil levels were positively associated with mortality due to cancer of the buccal cavity and pharynx, cancer of the esophagus, non-Hodgkin’s lymphoma, and breast cancer. These risks were evident only in women, and no association was found in men. To note, Cr in topsoil exists in the trivalent state, as was mentioned in the introduction; nevertheless, once it enters other environmental compartments, it may be oxidized to Cr(VI). 

Regarding the quality of the studies included within the review, all but a few are of high quality, as evaluated according to their design (Newcastle–Ottawa scale, 8–10 stars). Exposures and health outcomes were assessed via validated standardized procedures, appropriate statistical methods were employed, and controlling for potential confounders and covariates took place. Only six studies were scored with six or seven stars, which characterizes them of good quality as well.

## 4. Discussion

Not unreasonably, chromium has been characterized as a “double-edged sword”. This review comes to justify the delineation. Is ingested chromium an essential metal for physiological function? Or is it a toxic hazardous substance even when ingested? Does it have an oxidative or an anti-oxidative action? It was around 1936 when the German health authorities recognized that chromate dust exposure causes occupational lung cancer. It was in 1981 when Tor Noserth [96] published his review about the carcinogenicity of chromium in Environmental Health Perspectives. In that review, he writes: “The most important problems at present are whether trivalent chromium compounds cause cancer, and whether there is a difference in cancer causing effects between the soluble and the slightly soluble hexavalent compounds in the practical exposure situation. Dose estimates for risk estimation based on epidemiological investigations are also lacking”. For more than 50 years, the scientific community has been struggling to answer these questions. A plethora of research, different study designs, and diverse health outcomes have been published in an effort to elucidate the mechanisms and true nature of chromium’s interaction with biological tissues. 

The toxicity and carcinogenicity of hexavalent chromium when exposure occurs via inhalation is well established. In the present review, we compiled the literature of human studies on oral exposure to Cr(VI) and adverse health outcomes. Four areas have been identified that have been documented to carry elevated environmental levels of Cr(VI) in the water compartment. The first is the Jinzhou suburbs in Liaoning province, China, where the population has been exposed to extremely high, but not accurately determined, levels of Cr(VI) for more than 60 years. People in this region have been serving as the study population in many epidemiological studies up to now. Despite the conflicting results, the risks of lung and stomach cancer and the calculated SMRs cannot be overlooked. Similar to Jinzhou’s pollution load is that in Kanpur, India, where health complaints and symptoms have been recorded. Finally, environmental levels of Cr(VI) in water are at least 100 times lower in the exposed area in Greece of the prefecture of Voiotia, where elevated risks for liver and genitourinary organ cancers have been reported, along with urogenital infections and biochemical alterations. 

Nevertheless, the majority of recent research deals with biomonitoring studies, which in most cases are cross-sectional in design. Although biomonitoring studies excel at assessing exposure by determining the actual amount in the body, they do not provide information on the route of exposure. According to this, the concentrations of Cr in blood, urine, and tissue samples (hair, toenails) reported in the reviewed studies could be the result of Cr intake, either in trivalent or hexavalent form, through air, water, food, soil, or skin absorption. Studies that have monitored environmental levels of oral Cr exposure and related them to the internal Cr dose are quite helpful in delineating the source-to-body pathway. Furthermore, biomonitoring data alone do not provide information about the clinical significance of potential elevated values, because elevated concentrations do not necessarily imply significant health concerns.

Reference values for unexposed general populations or threshold values derived from toxicological or epidemiological data are required for meaningful interpretation of biomonitoring levels determined in the populations studied. It is now known that Cr in blood is an internal marker of Cr(VI) exposure, considering that the red blood cell membrane is permeable to Cr(VI) but not to Cr(III) [12,97,98]. Blood Cr concentrations vary by matrix (whole blood, plasma, or serum). The reviewed studies included healthy adult populations who served either as controls in case control studies or unexposed in cross-sectional and cohort studies. Blood chromium measured in these populations ranged from non-detectable levels to 0.92 μg/L, with median values from 0.15 μg/L to 0.42 μg/L [29,61,79], consistent with reference values reported to range from 0.01 to 1.2 μg/L [99,100]. It should be noted that a Chinese study involved a relatively large sample of 11,037 adults, representative of the general Chinese population [61]. The exposed population in the historically polluted area of Jinzhou city in China showed a median blood Cr value of 0.88 μg/L [67], not significantly different from other populations. However, serum Cr levels had median values of 1.66 μg/L [78] and 2.05 μg/L in [68], whereas the 75th percentiles reached 2.18 μg/L [78], 2.40 μg/L [89], and 4.98 μg/L [68]. Reference values for serum Cr have been reported to vary from 0.04 μg/L to 0.48 μg/L [99,100], much lower than those reported in the Chinese studies.

Although urine has become a traditional biomarker for assessing Cr exposure, it should be noted that it is not specific for Cr(VI) and reflects exposure to both Cr(III) and Cr(VI). The biological half-life of Cr in urine is very short, about 2 days [12,21]. Thus, Cr determinations in urine represent recent exposure. The 75th percentiles of urinary Cr distribution in the unexposed or control general populations included in the studies within this review extended from 1.12 μg/L [61] to 3.02 [84]. Urinary Cr reference values in the general unexposed population have been reported to range from 0.04 to 1.5 μg/L [100,101]. Notably, two Chinese studies conducted on presumably unexposed populations reported high urinary Cr concentrations, namely, P75: 45.12 μg/L [83] and P75: 36.25 [86], which are not comparable to other unexposed populations. Regarding the exposed population in Jianzhou province, China, urinary Cr levels differed from the rest of the studied populations, as the 75th percentiles were found to vary from 4.76 to 5.70 μg/L [65,66,77], possibly as an indicator of the high exposure of this population. For comparison purposes only, the French biological limit value (BLV) of 2.5 μg/L set for occupational exposure to Cr(VI) is reported, along with the 95th percentile of values determined in unexposed control workers (0.44 μg/g creatinine), in the EU human biomonitoring study (HBM4EU) [102].

Finally, tissues such as hair or toenails have the advantage of simple and non-invasive sampling and provide information about long-term exposure. Reference values for Cr in hair have been reported from 0.05 μg/g to 0.53 μg/g [103,104]. These levels are similar to those reported in this review (P75: 0.46 μg/g [29], P75: 0.12 μg/g [78]), whereas in [71], the mean values were 0.25–0.59 μg/g. A promising approach to overcoming the limitations of biomarkers is to use multiple internal exposure matrices and assess their relationship. Following this, a comprehensive picture of the biologically active dose can be obtained, and unravelling of the correlation of certain health effects with certain levels of exposure can be achieved. 

In terms of health outcomes, among the most studied is the impact of fetal exposure to Cr in utero. The ability of metals to cross the placental barrier, induce epigenetic modifications in the genome, or act as endocrine disruptors can adversely affect normal fetal development [35,50]. Seven studies examined the effect of maternal internal Cr dose on fetal development, and in five of them, with a total study population of 6134 mother–infant pairs, a significant decrease in either birth weight or length of the newborn was observed [36,38,40,41,42]. These prenatal effects appear to be sex-related, maybe due to different hormone levels during perinatal development and the estrogen disruptive function of chromium [45,58]. Shorter gestational age and risk of preterm delivery is another health outcome related to Cr exposure in utero, as observed in the two large birth cohorts [44,45] with a total of 12,698 mother–infant pairs. In addition to the fetus, exposure to Cr during pregnancy appears to also affect the mother [51,53,54]. Another critical window of development is early childhood, when metabolic rates are extremely high, and exposures may determine susceptibility to disease in adulthood [57,58]. The other face of chromium becomes apparent in [56], where a protective role against hypertension in preschool children was documented. This protective role, however, is not confirmed in adult populations [29,61,62,64].

It seems that oxidative stress is a major pathway for Cr to cause deleterious alterations during adulthood. Several studies have documented that elevated Cr levels in blood, urine, or hair are associated with alterations in either markers of normal liver function (total proteins, albumin, aminotransferases) [29,62,65] or markers of oxidative stress and lipid peroxidation (MDA, GSH-Px, SOD, and CAT) [27,67,73]. Exposure to chromium may also exacerbate lipid metabolism [29,62,66,67] and physiological renal function [72,79,84], although the findings are inconsistent [69,80,81,83]. Conflicting results have also been reported on the association, if any, of internal Cr levels with glucose levels and risk of diabetes. Some studies found no association [63,70], others found an increasing trend for diabetes [72], and two large USA cohort studies reported a lower risk of diabetes with the use of chromium supplements [74] or in subjects with detectable amounts of Cr in the urine [75]. 

Finally, cancer biomonitoring studies reveal possible implications of Cr in urothelial [91], thyroid [92], and skin cancers [90]. The underlying mechanisms have been reported to involve the detoxification process that Cr(VI) undergoes within the cell, a step-wise reduction through which highly reactive intermediate species -Cr(V), Cr(IV)-, and ROS are generated. These intermediate species and their end-product—Cr(III)—can cause DNA damage and other cellular insults. In vitro studies have found that Cr-DNA binary adducts can be repaired if relevant mechanisms are not disrupted. However, lasting damage may be caused by Cr coupled to the ROS scavengers. These large adducts (GSH-Cr-DNA, ascorbate-Cr-DNA, Cr-protein) can block replication and lead to mutation [105]. 

In real life, people are exposed not only to Cr but also to a variety of metals and other potentially hazardous substances. Consequently, advanced statistical methods should be applied to investigate the effects of simultaneous exposures. One promising approach to processing data of this kind is the Bayesian kernel machine regression model, which outstands conventional regression models in some respects, as it can explore overall effects of mixtures and elucidate interactions between components. Since it provides non-linear relationships, it better reflects the real nature of the interaction between chemicals and biological tissues. It determines the response function of a single component of the mixture while better dealing with possible confounders and collinearity than traditional regressions [46]. However, a major limitation of BKMR is that when exploring the dose–response relationship of a single component, it assumes that exposure to the other mixture components occurs at constant levels, leading to a potential bias. This assumption does not accurately reflect the actual situation, as people are exposed simultaneously to multiple chemicals at varying levels [48]. 

## 5. Conclusions and Future Directions

Oral exposure of the general population to Cr(VI) has been associated with adverse health effects in biomonitoring and epidemiological studies. Prenatal exposure may hinder proper fetal development and cause preterm delivery or low birth weight. Exposure to Cr later in life appears to alter specific physiological functions and is involved in metabolic pathways. Most of the studies are cross-sectional and do not allow causal inference; however, a few large cohort studies that have been conducted found no significant harmful effects, except for one that reported an elevated risk of skin cancer. Undoubtedly, there are contradictory findings that do not yet allow for firm conclusions. The biological role of chromium has been under scrutiny for many years. It is time to leave associations behind and search for causal inferences. Future research should focus on well-designed, large prospective cohorts and be conducted in the areas documented to exhibit elevated environmental levels of Cr(VI). Epidemiological studies should be accompanied by accurate and systematic monitoring of environmental levels of both Cr species, along with biomonitoring studies involving multiple biomarkers of exposure and effect. Under this condition, the entire pathway from the source to the body and from early health changes to disease will be revealed, and the exact biological roles of trivalent and hexavalent chromium will be discovered. This is the way to understand the potential adverse effects related to low levels of environmental exposure to chromium.

## Figures and Tables

**Table 4:** Summary of the studies on cancer risks being associated with Cr exposure.

Study Characteristics (Area, Time, Population)	Study Design	Exposure Variables	Biomonitoring Data [(Mean± SD) or Median]	Key Findings on Health Outcomes	Ref.
Mexico 2007–2011 452 breast cancer cases 439 controls	Case control	11 metals in urine (Al, As, Cd, Cr, Ni, Pb, Sb, Co, Mo, Sn, V)	Cr-U: 3.16 μg/g creatinine (P10: 1.27 μg/g, P90: 8.93 μg/g) (cases) Cr-U: 3.00 μg/g creatinine (P10: 1.35 μg/g, P90: 7.97 μg/g) (controls)	No difference of Cr levels in cases and controls (*p*_trend_ = 0.709) Breast cancer: mixture of Cr, Ni, Sb, Al, Pb, Sn **OR = 1.15 (95% CI: 1.06, 1.25)**	[88]
Italy 1993–1998 150 breast cancer cases 150 controls	Nested case control	6 metals in serum (Cd, Co, Cr, Mn, Pb, Tl)	Cr-S: P75: 2.10 μg/L (cases) Cr-S: P75: 2.40 μg/L (controls)	Breast cancer: no difference in Cr levels between cases and controls (*p* > 0.050) OR = 0.66 (95% CI: 0.26, 1.67) for Cr-S 0.63–2.40 μg/L vs. <0.50 μg/L OR = 0.60 (95% CI: 0.20, 1.82) for Cr-S >2.50 μg/L vs. <0.50 μg/L	[89]
USA 1984–2012 10,438 adults 6708 women 3730 men	Prospective cohort	5 metals in toenails (Cr, Fe, Hg, Se, Zn)	Cr-Toenails: 0.86 ± 2.83 μg/g (women) Cr-Toenails: 0.89 ± 1.82 μg/g (men)	**BCC: HR = 1.32 (95% CI: 1.12, 1.56)** (only in women) for Q5 (median Cr: 1.78 μg/g) vs. Q1 (median Cr: 0.12 μg/g) SCC: HR = 1.41 (95% CI 0.92, 2.15) (women) for Q4 (median Cr: 0.71 μg/g) vs. Q1 (median Cr: 0.12 μg/g) Melanoma: HR = 1.41 (95% CI: 0.92, 2.15) (women) Melanoma: HR = 0.95 (95% CI: 0.59, 1.54) (men)	[90]
Taiwan 2011–2013 205 urothelial carcinoma cases, 406 controls	Case control	5 metals in urine (Cr, As, Cd, Ni, Pb)	Cr-U: 0.61 ± 0.10 μg/g creatinine (mean ±SE) (controls) Cr-U: 2.38 ± 0.41 μg/g creatinine (mean ±SE) (patients)	**Urothelial cancer**: **OR = 2.01 (95% CI: 1.12, 3.60)** (Cr-U: 0.18–0.45 μg/g creatinine) **OR = 5.78 (95% CI: 3.37, 9.90)** (Cr-U: > 0.45 μg/g creatinine)	[91]
China 2017–2019 608 papillary thyroid cancer cases (74.7% females)	Patient study	10 elements in urine (Co, Cr, Cu, Fe, Mn, Mo, Se, Sr, Zn, and I)	Cr-U: 43.23 μg/g creatinine (P25: 21.61 μg/g, P75: 57.58 μg/g)	Clinicopathologic characteristics of papillary thyroid cancer **OR = 1.68, (95% CI 1.02, 2.77)** (Q4 vs. Q1) for multifocality, in single -element model No association in multi-element model	[92]
USA 2000–2014 2092 patients with pancreatic ductal adenocarcinoma 2353 controls	Case control	Self-reported exposure	Not determined	Pancreatic cancer: OR = 1.42, (95% CI 0.89, 2.26) for self-reported Cr exposure	[93]
Tunisia 2007–2010 49 bladder cancer cases 36 controls	Case control	Cr in tissues	Cr: 1.50 ± 3.23 μg/g (mean ± SE) in cancer tissues Cr: 10.09 ± 18.16 μg/g (mean ± SE) in adjacent tissues Cr: 13.48 ± 19.90 μg/g (mean ± SE) in control tissues	Significantly lower Cr level in cancer tissues	[94]
Spain 1999–2008	Ecological mortality	Exposure surrogate: Cr topsoil levels in 21,187 samples	Cr-Soil: 23.2 mg/kg (P25:15.6 mg/kg, P75: 33.8 mg/kg) (range: 0.50–2100 mg/kg)	Cancer of the buccal cavity and pharynx **RR = 1.149, (95% CI 1.036, 1.274)** (women only) Cancer of esophagus **RR = 1.328, (95% CI: 1.146, 1.544)** (women) Non-Hodgkin’s lymphoma: **RR = 1.092 (95% CI: 1.018, 1.170)** (women) Breast cancer: **RR = 1.045 (95% CI: 1.009, 1.082)** (women)	[95]

CI: confidence interval, Cr-S: Cr in serum, Cr-U: Cr in urine, HR: hazard ratio, OR: odds ratio, P25: 25th percentile, P75: 75th percentile, Q: quartile, RR: rate ratio, SE: standard error. Significant associations are indicated in bold.

## References

[1] Cardarelli F. (2008). Materials Handbook: A Concise Desktop Reference.

[2] International Agency for Research on Cancer (2012). Arsenic, metals, fibres, and dusts. A review of human carcinogens. IARC Monographs of the Evaluation of Carcinogenic Risks to Humans.

[3] Koleli N., Demir A., Prasad M.N.V., Shih K. (2016). Chromite. Environmental Materials and Waste.

[4] Kimbrough D.E., Cohen Y., Winer A.M., Creelman L., Mabuni C. (1999). A critical assessment of chromium in the environment. Crit. Rev. Environ. Sci. Technol..

[5] Fantoni D., Brozzo G., Canepa M., Cipolli F., Marini L., Ottonello G., Zuccolini M. (2002). Natural hexavalent chromium in groundwaters interacting with ophiolitic rocks. Environ. Geol..

[6] Levy L.S., Martin P.A., Bidstrup P.L. (1986). Investigation of the potential carcinogenicity of a range of chromium containing materials on rat lung. Br. J. Ind. Med..

[7] Hathaway J.A. (1989). Role of epidemiologic studies in evaluating the carcinogenicity of chromium compounds. Sci. Total Environ..

[8] DesMarais T.L., Costa M. (2019). Mechanisms of Chromium-Induced Toxicity. Curr. Opin. Toxicol..

[9] DeFlora S. (2000). Threshold mechanisms and site specificity in chromium (VI) carcinogenesis. Carcinogenesis.

[10] Zhitkovich A. (2011). Chromium in drinking water: Sources, metabolism, and cancer risks. Chem. Res. Toxicol..

[11] Sun H., Brocato J., Costa M. (2015). Oral Chromium Exposure and Toxicity. Curr. Environ. Health Rep..

[12] Kerger B.D., Paustenbach D.J., Corbett G.E., Finley B.L. (1996). Absorption and elimination of trivalent and hexavalent chromium in humans following ingestion of a bolus dose in drinking water. Toxicol. Appl. Pharmacol..

[13] Stern A.H. (2010). A quantitative assessment of the carcinogenicity of hexavalent chromium by the oral route and its relevance to human exposure. Environ. Res..

[14] EFSA NDA Panel (EFSA Panel on Dietetic Products, Nutrition and Allergies) (2014). Scientific Opinion on Dietary Reference Values for chromium. EFSA J..

[15] Vincent J.B. (2000). The biochemistry of chromium. J. Nutr..

[16] Chen G., Liu P., Pattar G.R., Tackett L., Bhonagiri P., Strawbridge A.B., Elmendorf J.S. (2006). Chromium activates glucose transporter 4 trafficking and enhances insulin-stimulated glucose transport in 3T3-L1 adipocytes via a cholesterol-dependent mechanism. Mol. Endocrinol..

[17] Verdonck J., Duca R.C., Galea K.S., Iavicoli I., Poels K., Töreyin Z.N., Vanoirbeek J., Godderis L. (2021). Systematic review of biomonitoring data on occupational exposure to hexavalent chromium. Int. J. Hyg. Environ. Health.

[18] Page M.J., McKenzie J.E., Bossuyt P.M., Boutron I., Hoffmann T.C., Mulrow C.D., Shamseer L., Tetzlaff J.M., Akl E.A., Brennan S.E. (2021). The PRISMA 2020 statement: An updated guideline for reporting systematic reviews. BMJ.

[19] Wells G.A., Shea B., O’Connell D., Peterson J., Welch V., Losos M., Tugwell P. The Newcastle-Ottawa Scale (NOS) for Assessing the Quality If Nonrandomized Studies in Meta-Analyses. http://www.ohri.ca/programs/clinical_epidemiology/oxford.htm.

[20] Modesti P.A., Reboldi G., Cappuccio F.P., Agyemang C., Remuzzi G., Rapi S., Perruolo E., Parati G., ESH Working Group on CV Risk in Low Resource Settings (2016). Panethnic Differences in Blood Pressure in Europe: A Systematic Review and Meta-Analysis. PLoS ONE.

[21] ATSDR (Agency for Toxic Substances and Disease Registry) (2012). Toxicological Profile for Chromium. US Department of Health and Human Services, Atlanta. https://www.atsdr.cdc.gov/ToxProfiles/tp7.pdf.

[22] Zhang J.D., Li X.L. (1987). Chromium pollution of soil and water in Jinzhou. Zhonghua Yu Fang Yi Xue Za Zhi.

[23] Zhang J.D., Li S. (1997). Cancer mortality in a Chinese population exposed to hexavalent chromium in water. J. Occup. Environ. Med..

[24] Beaumont J.J., Sedman R.M., Reynolds S.D., Sherman C.D., Li L.H., Howd R.A., Sandy M.S., Zeise L., Alexeeff G.V. (2008). Cancer mortality in five villages in China with hexavalent chromium-contaminated drinking water. Epidemiology.

[25] Kerger B.D., Butler W.J., Paustenbach D.J., Zhang J., Li S. (2009). Cancer mortality in Chinese populations surrounding an alloy plant with chromium smelting operations. J. Toxicol. Environ. Health Pt A.

[26] Smith A.H. (2008). Hexavalent chromium, yellow water, and cancer: A convoluted saga. Epidemiology.

[27] Xu J., Zhao M., Pei L., Zhang R., Liu X., Wei L., Yang M., Xu Q. (2018). Oxidative stress and DNA damage in a long-term hexavalent chromium-exposed population in North China: A cross-sectional study. BMJ Open.

[28] Sharma P., Bihari V., Agarwal S.K., Verma V., Kesavachandran C.N., Pangtey B.S., Mathur N., Singh K.P., Srivastava M., Goel S.K. (2012). Groundwater contaminated with hexavalent chromium (Cr(VI)): A health survey and clinical examination of community inhabitants (Kanpur, India). PLoS ONE.

[29] Sazakli E., Villanueva C.M., Kogevinas M., Maltezis K., Mouzaki A., Leotsinidis M. (2014). Chromium in drinking water: Association with biomarkers of exposure and effect. Int. J. Environ. Res. Public Health.

[30] Karagiannis D., Deliveliotis C., Papadimitriou E., Riza E., Lykou A., Petralias A., Papatsoris A., Linos A. (2015). Oral exposure to hexavalent chromium through drinking water and urologic morbidity in an industrial area of Greece. J. Public Health.

[31] Linos A., Petralias A., Christophi C.A., Christoforidou E., Kouroutou P., Stoltidis M., Veloudaki A., Tzala E., Makris K.C., Karagas M.R. (2011). Oral ingestion of hexavalent chromium through drinking water and cancer mortality in an industrial area of Greece—An ecological study. Environ. Health.

[32] California EPA Office of Environmental Health Hazard Assessment (OEHHA) Public Health Goal for Hexavalent Chromium (CrVI) in Drinking Water 2011. https://oehha.ca.gov/media/downloads/water/chemicals/phg/cr6phg072911.pdf.

[33] Fryzek J.P., Mumma M.T., McLaughlin J.K., Henderson B.E., Blot W.J. (2001). Cancer mortality in relation to environmental chromium exposure. J. Occup. Environ. Med..

[34] Lacagnina S. (2019). The Developmental Origins of Health and Disease (DOHaD). Am. J. Lifestyle Med..

[35] Darney S., Fowler B., Grandjean P., Heindel J., Mattison D., Slikker W. (2011). Prenatal Programming and Toxicity II (PPTOX II): Role of environmental stressors in the developmental origins of disease. Reprod. Toxicol..

[36] Xia W., Hu J., Zhang B., Li Y., Wise J.P., Bassig B.A., Zhou A., Savitz D.A., Xiong C., Zhao J. (2016). A case-control study of maternal exposure to chromium and infant low birth weight in China. Chemosphere.

[37] Yang X., Li Y., Li J., Bao S., Zhou A., Xu S., Xia W. (2020). Associations between exposure to metal mixtures and birth weight. Environ. Pollut..

[38] Michael T., Kohn E., Daniel S., Hazan A., Berkovitch M., Brik A., Hochwald O., Borenstein-Levin L., Betser M., Moskovich M. (2022). Prenatal exposure to heavy metal mixtures and anthropometric birth outcomes: A cross-sectional study. Environ. Health.

[39] Cabrera-Rodríguez R., Luzardo O.P., González-Antuña A., Boada L.D., Almeida-González M., Camacho M., Zumbado M., Acosta-Dacal A.C., Rial-Berriel C., Henríquez-Hernández L.A. (2018). Occurrence of 44 elements in human cord blood and their association with growth indicators in newborns. Environ. Int..

[40] Freire C., Amaya E., Gil F., Murcia M., LLop S., Casas M., Vrijheid M., Lertxundi A., Irizar A., Fernández-Tardón G. (2019). Placental metal concentrations and birth outcomes: The Environment and Childhood (INMA) project. Int. J. Hyg. Environ. Health.

[41] Peng Y., Hu J., Li Y., Zhang B., Liu W., Li H., Zhang H., Hu C., Chen X., Xia W. (2018). Exposure to chromium during pregnancy and longitudinally assessed fetal growth: Findings from a prospective cohort. Environ. Int..

[42] Dou Y., Yin Y., Li Z., Du J., Jiang Y., Jiang T., Guo W., Qin R., Li M., Lv H. (2022). Maternal exposure to metal mixtures during early pregnancy and fetal growth in the Jiangsu Birth Cohort, China. Environ. Res..

[43] Yu Y., Gao M., Wang X., Guo Y., Pang Y., Yan H., Hao Y., Zhang Y., Zhang L., Ye R. (2019). Recommended acceptable levels of maternal serum typical toxic metals from the perspective of spontaneous preterm birth in Shanxi Province, China. Sci. Total. Environ..

[44] Pan X., Hu J., Xia W., Zhang B., Liu W., Zhang C., Yang J., Hu C., Zhou A., Chen Z. (2017). Prenatal chromium exposure and risk of preterm birth: A cohort study in Hubei, China. Sci. Rep..

[45] Huang S., Xia W., Li Y., Zhang B., Zhou A., Zheng T., Qian Z., Huang Z., Lu S., Chen Z. (2017). Association between maternal urinary chromium and premature rupture of membranes in the Healthy Baby Cohort study in China. Environ. Pollut..

[46] Tian T., Yin S., Jin L., Liu J., Wang C., Wei J., Liu M., Li Z., Wang L., Yin C. (2021). Single and mixed effects of metallic elements in maternal serum during pregnancy on risk for fetal neural tube defects: A Bayesian kernel regression approach. Environ. Pollut..

[47] Ou Y., Bloom M.S., Nie Z., Han F., Mai J., Chen J., Lin S., Liu X., Zhuang J. (2017). Associations between toxic and essential trace elements in maternal blood and fetal congenital heart defects. Environ. Int..

[48] Xu C., Xu J., Zhang X., Xu S., Liu Q., Weng Z., Gu A. (2021). Serum nickel is associated with craniosynostosis risk: Evidence from humans and mice. Environ. Int..

[49] Ruan F., Zhang J., Liu J., Sun X., Li Y., Xu S., Xia W. (2022). Association between prenatal exposure to metal mixtures and early childhood allergic diseases. Environ. Res..

[50] Vänskä M., Diab S.Y., Perko K., Quota S.R., Albarqouni N.M.A., Myöhänen A., Punamäki R.L., Manduca P. (2019). Toxic Environment of war: Maternal prenatal heavy metal load predicts infant emotional development. Infant Behav. Dev..

[51] Levin-Schwartz Y., Cowell W., Leon Hsu H.H., Enlow M.B., Amarasiriwardena C., Andra S.S., Wright R.J., Wright R.O. (2022). Metal mixtures are associated with increased anxiety during pregnancy. Environ. Res..

[52] Leal C.A., Schetinger M.R., Leal D.B., Morsch V.M., da Silva A.S., Rezer J.F., de Bairros A.V., Jaques J.A. (2011). Oxidative stress and antioxidant defenses in pregnant women. Redox Rep..

[53] Zhu G., Zheng T., Xia C., Qi L., Papandonatos G.D., Ming Y., Zeng Z., Zhang X., Zhang H., Li Y. (2021). Plasma levels of trace element status in early pregnancy and the risk of gestational diabetes mellitus: A nested case-control study. J. Trace Elem. Med. Biol..

[54] Bommarito P.A., Kim S.S., Meeker J.D., Fry R.C., Cantonwine D.E., McElrath T.F., Ferguson K.K. (2019). Urinary trace metals, maternal circulating angiogenic biomarkers, and preeclampsia: A single-contaminant and mixture-based approach. Environ. Health.

[55] Wu L.L., Gong W., Shen S.P., Wang Z.H., Yao J.X., Wang J., Yu J., Gao R., Wu G. (2017). Multiple metal exposures and their correlation with monoamine neurotransmitter metabolism in Chinese electroplating workers. Chemosphere.

[56] Liu Y., Yu L., Zhu M., Lin W., Liu Y., Li M., Zhang Y., Ji H., Wang J. (2022). Associations of exposure to multiple metals with blood pressure and hypertension: A cross-sectional study in Chinese preschool children. Chemosphere.

[57] Zhumalina A.K., Bekmukhambetov E.Z., Tusupkaliev B.T., Zharlikasinova M.B. (2019). Development of scientifically justified proposals on the prevention and treatment of environmentally determined constitutional growth delay in children in the West Kazakhstan region. Environ. Geochem. Health.

[58] Caparros-Gonzalez R.A., Giménez-Asensio M.J., González-Alzaga B., Aguilar-Garduño C., Lorca-Marín J.A., Alguacil J., Gómez-Becerra I., Gómez-Ariza J.L., García-Barrera T., Hernandez A.F. (2019). Childhood chromium exposure and neuropsychological development in children living in two polluted areas in southern Spain. Environ. Pollut..

[59] Quinteros F.A., Poliandri A.H., Machiavelli L.I., Cabilla J.P., Duvilanski B.H. (2007). In vivo and in vitro effects of chromium VI on anterior pituitary hormone release and cell viability. Toxicol. Appl. Pharmacol..

[60] Piñeiro X.F., Ave M.T., Mallah N., Caamaño-Isorna F., Jiménez A.N.G., Vieira D.N., Bianchini F., Muñoz-Barús J.I. (2021). Heavy metal contamination in Peru: Implications on children’s health. Sci. Rep..

[61] Qu Y., Lv Y., Ji S., Ding L., Zhao F., Zhu Y., Zhang W., Hu X., Lu Y., Li Y. (2022). Effect of exposures to mixtures of lead and various metals on hypertension, pre-hypertension, and blood pressure: A cross-sectional study from the China National Human Biomonitoring. Environ. Pollut..

[62] Liu X., Zhang D., Wu X., Tu J., Gong C., Li Y., Cui W., Chen J., Lu S. (2022). Urinary metals as influencing factors of coronary heart disease among a population in Guangzhou, China. Ecotoxicol. Environ. Saf..

[63] Son J., Morris J.S., Park K. (2018). Toenail Chromium Concentration and Metabolic Syndrome among Korean Adults. Int. J. Environ. Res. Public Health.

[64] Feng W., He X., Chen M., Deng S., Qiu G., Li X., Liu C., Li J., Deng Q., Huang S. (2015). Urinary metals and heart rate variability: A cross-sectional study of urban adults in Wuhan, China. Environ. Health Perspect..

[65] Zhao M., Ge X., Xu J., Li A., Mei Y., Yin G., Wu J., Liu X., Wei L., Xu Q. (2022). Association between urine metals and liver function biomarkers in Northeast China: A cross-sectional study. Ecotoxicol. Environ. Saf..

[66] Zhao M., Yin G., Xu J., Ge X., Li A., Mei Y., Wu J., Liu X., Wei L., Xu Q. (2023). Independent, combine and interactive effects of heavy metal exposure on dyslipidemia biomarkers: A cross-sectional study in northeastern China. Ecotoxicol. Environ. Saf..

[67] Xu J., Zhao M., Pei L., Liu X., Wei L., Li A., Mei Y., Xu Q. (2020). Effects of heavy metal mixture exposure on hematological and biomedical parameters mediated by oxidative stress. Sci. Total. Environ..

[68] Chang Z., Qiu J., Wang K., Liu X., Fan L., Liu X., Zhao Y., Zhang Y. (2023). The relationship between co-exposure to multiple heavy metals and liver damage. J. Trace Elem. Med. Bio.l.

[69] Xiao L., Zhou Y., Ma J., Cao L., Wang B., Zhu C., Yang S., Li W., Zhang Z., Wang D. (2019). The cross-sectional and longitudinal associations of chromium with dyslipidemia: A prospective cohort study of urban adults in China. Chemosphere.

[70] Liu L., Li A., Xu Q., Wang Q., Han F., Xu C., Liu Z., Xu D., Xu D. (2022). The association between urine elements and fasting glucose levels in a community-based elderly people in Beijing. Environ. Sci. Pollut. Res. Int..

[71] Bibi M., Hashmi M.Z., Malik R.N. (2016). The level and distribution of heavy metals and changes in oxidative stress indices in humans from Lahore district, Pakistan. Hum. Exp. Toxicol..

[72] Velmurugan G., Swaminathan K., Veerasekar G., Purnell J.Q., Mohanraj S., Dhivakar M., Avula A.K., Cherian M., Palaniswami N.G., Alexander T. (2018). Metals in urine in relation to the prevalence of pre-diabetes, diabetes and atherosclerosis in rural India. Occup. Environ. Med..

[73] Domingo-Relloso A., Grau-Perez M., Galan-Chilet I., Garrido-Martinez M.J., Tormos C., Navas-Acien A., Gomez-Ariza J.L., Monzo-Beltran L., Saez-Tormo G., Garcia-Barrera T. (2019). Urinary metals and metal mixtures and oxidative stress biomarkers in an adult population from Spain: The Hortega Study. Environ. Int..

[74] McIver D.J., Grizales A.M., Brownstein J.S., Goldfine A.B. (2015). Risk of Type 2 Diabetes Is Lower in US Adults Taking Chromium-Containing Supplements. J. Nutr..

[75] Wang X., Karvonen-Gutierrez C.A., Herman W.H., Mukherjee B., Harlow S.D., Park S.K. (2020). Urinary metals and incident diabetes in midlife women: Study of Women’s Health Across the Nation (SWAN). BMJ Open Diabetes Res. Care.

[76] Zhang R., Xiang Y., Ran Q., Deng X., Xiao Y., Xiang L., Li Z. (2014). Involvement of calcium, reactive oxygen species, and ATP in hexavalent chromium-induced damage in red blood cells. Cell. Physiol. Biochem..

[77] Zhao M., Ge X., Xu J., Li A., Mei Y., Zhao J., Zhou Q., Liu X., Wei L., Xu Q. (2022). Negatively interactive effect of chromium and cadmium on obesity: Evidence from adults living near ferrochromium factory. Ecotoxicol. Environ. Saf..

[78] Tinkov A.A., Skalnaya M.G., Ajsuvakova O.P., Serebryansky E.P., Chao J.C., Aschner M., Skalny A.V. (2021). Selenium, Zinc, Chromium, and Vanadium Levels in Serum, Hair, and Urine Samples of Obese Adults Assessed by Inductively Coupled Plasma Mass Spectrometry. Biol. Trace Elem. Res..

[79] Chung M.C., Hsu H.T., Mao Y.C., Wu C.C., Ho C.T., Liu C.S., Chung C.J. (2022). Association and mediation analyses among multiple metals exposure, plasma folate, and community-based impaired estimated glomerular filtration rate in central Taiwan. Environ. Health.

[80] Tsai T.L., Kuo C.C., Pan W.H., Chung Y.T., Chen C.Y., Wu T.N., Wang S.L. (2017). The decline in kidney function with chromium exposure is exacerbated with co-exposure to lead and cadmium. Kidney Int..

[81] Tsai C.C., Wu C.L., Kor C.T., Lian I.B., Chang C.H., Chang T.H., Chang C.C., Chiu P.F. (2018). Prospective associations between environmental heavy metal exposure and renal outcomes in adults with chronic kidney disease. Nephrology.

[82] Yang F., Yi X., Guo J., Xu S., Xiao Y., Huang X., Duan Y., Luo D., Xiao S., Huang Z. (2019). Association of plasma and urine metals levels with kidney function: A population-based cross-sectional study in China. Chemosphere.

[83] Liu Y., Zhang C., Qin Z., Yang Q., Lei J., Tang X., Wang Q., Hong F. (2022). Analysis of Threshold Effect of Urinary Heavy Metal Elements on the High Prevalence of Nephrolithiasis in Men. Biol. Trace Elem. Res..

[84] Liu S., Zhang L., Luo N., Wang M., Tang C., Jing J., Chen H., Hu Q., Tan L., Ma X. (2023). Metal mixture exposure and the risk for immunoglobulin A nephropathy: Evidence from weighted quantile sum regression. Environ. Sci. Pollut. Res. Int..

[85] Branch F.M., Perry M.J., Chen Z., Louis G.M.B. (2021). Metal(loid)s and human semen quality: The LIFE Study. Reprod. Toxicol..

[86] Li Y.Q., Wang Q., Liu R., Li G.A., He J.L., Huang F., Zhou Y.F. (2023). Associations of exposure to multiple metals with the risk of age-related cataract in Anhui, China: A case-control study. Environ. Sci. Pollut. Res. Int..

[87] Sánchez-Díaz G., Escobar F., Badland H., Arias-Merino G., Posada de la Paz M., Alonso-Ferreira V. (2018). Geographic Analysis of Motor Neuron Disease Mortality and Heavy Metals Released to Rivers in Spain. Int. J. Environ. Res. Public Health.

[88] Mérida-Ortega Á., Rothenberg S.J., Cebrián M.E., López-Carrillo L. (2022). Breast cancer and urinary metal mixtures in Mexican women. Environ. Res..

[89] Caini S., Cozzolino F., Saieva C., Aprea M.C., De Bonfioli Cavalcabo’ N., Ermini I., Assedi M., Biagiotti D., Trane C., Facchini L. (2023). Serum heavy metals and breast cancer risk: A case-control study nested in the Florence cohort of the EPIC (European Prospective Investigation into Cancer and nutrition) study. Sci. Total. Environ..

[90] Matthews N.H., Koh M., Li W.Q., Li T., Willett W.C., Stampfer M.J., Christiani D.C., Morris J.S., Qureshi A.A., Cho E. (2019). A Prospective Study of Toenail Trace Element Levels and Risk of Skin Cancer. Cancer Epidemiol. Biomark. Prev..

[91] Chang C.H., Liu C.S., Liu H.J., Huang C.P., Huang C.Y., Hsu H.T., Liou S.H., Chung C.J. (2016). Association between levels of urinary heavy metals and increased risk of urothelial carcinoma. Int. J. Urol..

[92] Hu M.J., He J.L., Tong X.R., Yang W.J., Zhao H.H., Li G.A., Huang F. (2021). Associations between essential microelements exposure and the aggressive clinicopathologic characteristics of papillary thyroid cancer. Biometals.

[93] Antwi S.O., Eckert E.C., Sabaque C.V., Leof E.R., Hawthorne K.M., Bamlet W.R., Chaffee K.G., Oberg A.L., Petersen G.M. (2015). Exposure to environmental chemicals and heavy metals, and risk of pancreatic cancer. Cancer Causes Control.

[94] Feki-Tounsi M., Olmedo P., Gil F., Mhiri M.N., Rebai A., Hamza-Chaffai A. (2014). Trace metal quantification in bladder biopsies from tumoral lesions of Tunisian cancer and controls subjects. Environ. Sci. Pollut. Res. Int..

[95] Núñez O., Fernández-Navarro P., Martín-Méndez I., Bel-Lan A., Locutura J.F., López-Abente G. (2016). Arsenic and chromium topsoil levels and cancer mortality in Spain. Environ. Sci. Pollut. Res. Int..

[96] Norseth T. (1981). The carcinogenicity of chromium. Environ. Health Perspect..

[97] Alexander J., Aaseth J. (1995). Uptake of chromate in human red blood cells and isolated rat liver cells: The role of the anion carrier. Analyst.

[98] Qu Q., Li X., An F., Jia G., Liu L., Watanabe-Meserve H., Koenig K., Cohen B., Costa M., Roy N. (2008). CrVI exposure and biomarkers: Cr in erythrocytes in relation to exposure and polymorphisms of genes encoding anion transport proteins. Biomarkers.

[99] Alimonti A., Bocca B., Mannella E., Petrucci F., Zennaro F., Cotichini R., D’Ippolito C., Agresti A., Caimi S., Forte G. (2005). Assessment of reference values for selected elements in a healthy urban population. Ann. Ist. Super Sanità.

[100] Minoia C., Sabbioni E., Apostoli P., Pietra R., Pozzoli L., Gallorini M., Nicolaou G., Alessio L., Capodaglio E. (1990). Trace element reference values in tissues from inhabitants of the European community. I. A study of 46 elements in urine, blood and serum of Italian subjects. Sci. Total. Environ..

[101] Morton J., Tan E., Leese E., Cocker J. (2014). Determination of 61 elements in urine samples collected from a non-occupationally exposed UK adult population. Toxicol. Lett..

[102] Santonen T., Porras S.P., Bocca B., Bousoumah R., Duca R.C., Galea K.S., Godderis L., Göen T., Hardy E., Iavicoli I. (2022). HBM4EU chromates study—Overall results and recommendations for the biomonitoring of occupational exposure to hexavalent chromium. Environ. Res..

[103] Skalny A.V., Skalnaya M.G., Tinkov A.A., Serebryansky E.P., Demidov V.A., Lobanova Y.N., Grabeklis A.R., Berezkina E.S., Gryazeva I.V., Skalny A.A. (2015). Hair concentration of essential trace elements in adult non-exposed Russian population. Environ. Monit. Assess..

[104] Rodushkin I., Ödman F., Branth S. (1999). Multielement analysis of whole blood by high resolution inductively coupled plasma mass spectrometry. Fresen. J. Anal. Chem..

[105] Chen Q.Y., DesMarais T., Costa M. (2019). Metals and Mechanisms of Carcinogenesis. Annu. Rev. Pharmacol. Toxicol..

